# BEESCOUT: A model of bee scouting behaviour and a software tool for characterizing nectar/pollen landscapes for BEEHAVE

**DOI:** 10.1016/j.ecolmodel.2016.09.013

**Published:** 2016-11-24

**Authors:** M.A. Becher, V. Grimm, J. Knapp, J. Horn, G. Twiston-Davies, J.L. Osborne

**Affiliations:** a Environment & Sustainability Institute, University of Exeter, Penryn Campus, Penryn, Cornwall TR10 9FE, UK; b UFZ, Helmholtz Centre for Environmental Research—UFZ, Permoserstr. 15, 04318 Leipzig, Germany; c German Centre for Integrative Biodiversity Research (iDiv) Halle-Jena-Leipzig, Deutscher Platz 5e, 04103 Leipzig, Germany

**Keywords:** Honeybee, Bumblebee, Searching, Flight pattern, Harmonic radar, Individual-based model

## Abstract

Social bees are central place foragers collecting floral resources from the surrounding landscape, but little is known about the probability of a scouting bee finding a particular flower patch. We therefore developed a software tool, BEESCOUT, to theoretically examine how bees might explore a landscape and distribute their scouting activities over time and space. An image file can be imported, which is interpreted by the model as a “forage map” with certain colours representing certain crops or habitat types as specified by the user. BEESCOUT calculates the size and location of these potential food sources in that landscape relative to a bee colony. An individual-based model then determines the detection probabilities of the food patches by bees, based on parameter values gathered from the flight patterns of radar-tracked honeybees and bumblebees. Various “search modes” describe hypothetical search strategies for the long-range exploration of scouting bees. The resulting detection probabilities of forage patches can be used as input for the recently developed honeybee model BEEHAVE, to explore realistic scenarios of colony growth and death in response to different stressors. In example simulations, we find that detection probabilities for food sources close to the colony fit empirical data reasonably well. However, for food sources further away no empirical data are available to validate model output. The simulated detection probabilities depend largely on the bees’ search mode, and whether they exchange information about food source locations. Nevertheless, we show that landscape structure and connectivity of food sources can have a strong impact on the results. We believe that BEESCOUT is a valuable tool to better understand how landscape configurations and searching behaviour of bees affect detection probabilities of food sources. It can also guide the collection of relevant data and the design of experiments to close knowledge gaps, and provides a useful extension to the BEEHAVE honeybee model, enabling future users to explore how landscape structure and food availability affect the foraging decisions and patch visitation rates of the bees and, in consequence, to predict colony development and survival.

## Highlights

• BEESCOUT is a spatially explicit, individual-based model of scouting bees.
• It determines the detection probabilities of food sources.
• It can be linked to the honey bee model BEEHAVE to predict colony development.

## Graphical abstract

**Figure:**
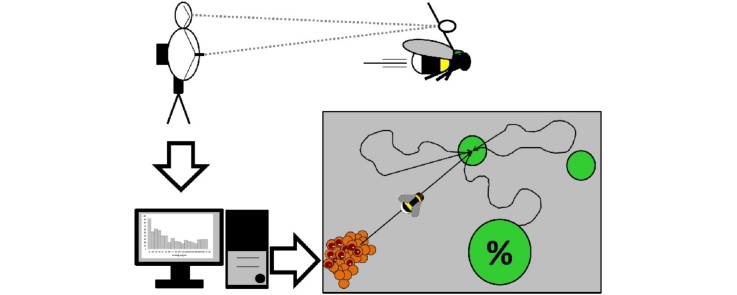

## 1 Introduction

Social bees are central place foragers who collect nectar and pollen from food sources in the surrounding landscape to store in their nest and feed to the brood (Michener, 1974, Pyke, 1984, Dukas and Edelstein-Keshet, 1998). The colonies’ efficiency to utilise food sources can be increased when foragers communicate their foraging success to nest-mates (Sherman and Visscher, 2002, Dornhaus and Chittka, 2004) thus reducing the need for individuals to scout. Consequently, the allocation of workers to either searching for or exploiting food sources is a crucial process which has been intensively investigated both empirically (Seeley, 1983, Biesmeijer and de Vries, 2001, Sumpter and Pratt, 2003, Osborne et al., 2013) and theoretically (see overview in Becher et al., 2013).

However, to exploit a food source, the bees need to find it in the first place. Although tracking of bees with harmonic radar is possible and provides valuable data on their flight patterns (Capaldi et al., 2000, Osborne et al., 2013), the operating range of this technique is limited and data collection is time consuming, as only one bee is tracked at a time. It therefore remains difficult to assess the probability of food patches being detected by colony members (Seeley, 1983, Beekman et al., 2007, Reynolds et al., 2007a). Knowledge of such detection probabilities, though, would not only be useful in itself but could also be applied to predict the bees' foraging success and food influx in a given landscape, and the pollination services provided by a colony.

To assess the likelihood of bees finding a certain food source not only the flight patterns have to be taken into account, but also some landscape features, as bees might avoid some areas (like water surfaces), use landmarks for orientation (Dyer, 1996, Wolf et al., 2014), or search specifically in areas where they have been feeding before (Reynolds et al., 2009).

We have therefore developed a software tool, BEESCOUT, to theoretically examine how bees might explore a landscape and distribute their scouting activities over time and space. The model was implemented in the free software platform NetLogo (Wilensky, 1999) and follows an individual-based approach. An image file, serving as a “forage map”, can be imported with certain areas being interpreted as crop/habitat fields or obstacles according to user-defined colours. Size, number and locations of the potential food sources are calculated and then the detection probability of each food source is determined based on the flight patterns of the bees. These flight patterns are based on the step lengths and turning angles from empirical flight tracks of individual scouting honeybees and bumble bees, recorded with the harmonic radar technique (Riley et al., 1996, Osborne et al., 2013), which allows the simulation of their spatial distribution in the landscape (Baars, 1979, Kindvall, 1999, Codling et al., 2008).

The model can be used for both honeybees and bumblebees which differ not only in their flight patterns but also in their recruitment strategy at the colony level. If information on the location of a food source is communicated to nest-mates (e.g. via waggle dances in honeybees) this may lead to higher detection probabilities and faster acquisition of knowledge about the spatial arrangement of food sources in the landscape (Dornhaus and Chittka, 2004, Grüter and Farina, 2009). BEESCOUT therefore includes the option to compare the patch detection success of scouting strategies for bees that communicate different levels of information about the landscape using different “search modes”. It should be noted, though, that little is known about where in a landscape scouting bees go after leaving their colony. They might immediately start random search, or first fly directly to a certain point in the landscape (“field destination”) that they know or have learned about from other bees, or a combination of both, depending on the spatio-temporal dynamics of flower patches, and on the bee species considered. BEESCOUT thus offers choices between using pure and mixed selections, which allow users to learn how sensitive detection probabilities are to characteristics of landscape, species, and search modes. Knowledge of food source detection is not only useful in the context of foraging bees, but we have even less empirical evidence of scouting and recruitment patterns in other Central Place Foragers, such as birds (Pinaud and Weimerskirch, 2005, Wakefield et al., 2013), and modelling their behavioural response to landscapes with patchy resources (Lima and Zollner, 1996) is likely to be insightful for monitoring the effects of environmental change and fragmentation on foraging efficiency and ultimately survival.

BEESCOUT was designed in a way that allows it to be linked with the honeybee model BEEHAVE (Becher et al., 2014). BEEHAVE simulates the development of a single honey bee colony as a consequence of the foraging success of worker bees in a heterogeneous landscape, and to that end it is being used to assess the effects of multiple in hive and landscape stressors on honeybee colony survival (EFSA, 2015, Horn et al., 2015, Rumkee et al., 2015, McMahon et al., 2016). The BEESCOUT landscape module can be used to create input files for BEEHAVE. For this purpose, additional options are provided to allow the user to specify nectar and pollen production of habitat and crop types (although this does not affect the BEESCOUT model itself, where food collection is not addressed). With this new landscape module we are now able to fill the gap identified by the European Food Safety Authority (EFSA, 2015) in their evaluation of the suitability of BEEHAVE for use in a regulatory context and for risk assessment. In their conclusion they recommended that BEEHAVE should form the basis for evaluating the effects of multiple stressors on honeybees at the landscape scale, but that the representation of the landscape needed further development since BEEHAVE itself has limited capacity for the definition of complex nectar and pollen landscapes (such as those illustrated by Baude et al., 2016).

In the following, we describe BEESCOUT and the underlying data. We also present initial results: comparing simulations with empirical data on scouting bees and examining with the model how forage patch detection is affected by “search mode” and landscape configuration. The Supplementary material includes the NetLogo program implementing BEESCOUT (S1), the input files for the example simulations, a detailed model description (S2), and a manual (S3).

## 2 Materials and methods

### 2.1 The BEESCOUT software tool

A detailed description of BEESCOUT, following the ODD (Overview, Design concepts, Details) protocol (Grimm et al., 2006, Grimm et al., 2010), is provided in the Supplementary material (S2), together with a list of all state variables (S4), input maps (S6), an exemplary sensitivity analysis (S10), an user manual (S3), and the model itself (S1). We highly recommend prospective users to make themselves familiar with the Supplementary material when using the software tool. BEESCOUT is licensed under the GNU General Public License (Supplementary material S7).

**Fig. 1:**
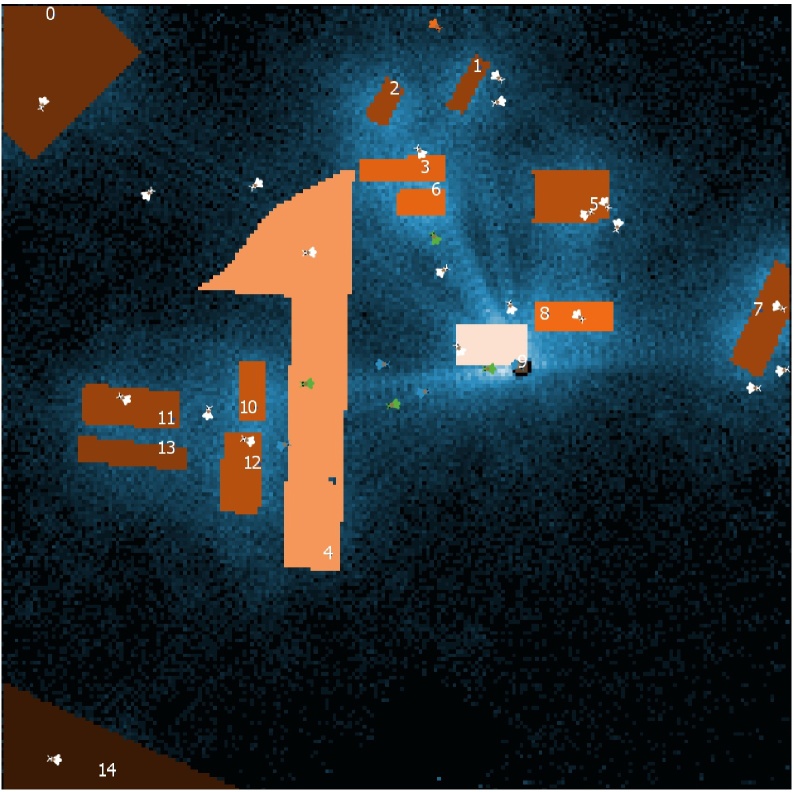
Screenshot of the model world (landscape) of BEESCOUT interface, using the settings of Simulation 1 (see Results section) with 30 bumblebee scouts exploring a 700 × 700 m landscape. Lighter background areas relate to higher bee visiting densities and lighter shaded flower patches indicate a higher detection probability. Bees of different colours are in a different ‘flight phase’: green (phase 1): the bee is heading in a straight vector flight towards a field destination (e.g. a flower patch already visited); white (phase 2): the bee is in a small scale flight pattern; orange (phase 2): the bee is “looping”, i.e. returns to its field destination; blue (phase 3): the bee returns to the colony in a straight vector flight. Patches can be detected in any flight phase. (For interpretation of the references to colour in this figure legend, the reader is referred to the web version of this article.)

The model includes the following entities:

1) The world (dimensions: 300 × 210 Netlogo grid cells), in which bees can search for food sources (“flower patches”, often referred to just as “patches” in the following). It is based on a forage map, where the user specifies patches where nectar and/or pollen are available in flowers. The map is either imported by the user as an image file (supported formats: BMP, JPG, GIF, and PNG), or an artificial (stylized) landscape can be generated within the program. If bees reach the borders of the world, they bounce back and randomly change their direction. To avoid a strong edge effect, we recommend using a sufficiently large map in relation to the simulated scouting period and search mode (the maximal displacement values in Table 1 may serve as a guideline). The scaling of the world depends on the scaling of the forage map. Scaling is incorporated into the model by the user, relating the distance between two grid cells in the modelled world to the real distance represented.
2) A certain number of flower patches, which represent fields with crops or habitats with wild flowers. A flower patch is defined by the model if there is a contiguous area of Netlogo grid cells of the same colour on the map. It is possible to distinguish four different patch types using four colours (red, green, yellow and blue). The user chooses which of these four colours are to be considered as food sources and, optionally, what crop or habitat type a colour represents. A “detection” of a flower patch takes place when a bee encounters any grid cell belonging to that flower patch for the first time during its current scouting trip. In realistic conditions, a bee will detect a flower patch depending on visual and olfactory cues, but it is unclear at what range these cues are active, so the model is currently conservative in assuming no actual detection until the bee is in the vicinity of the patch. From the number of detections and the total number of scouting trips of all bees, the detection probabilities per trip can be calculated for each flower patch. The detection probabilities are *not* dependent on rewards in the flower patch in this model and there is no exploitation of food sources taking place in BEESCOUT (the option to define nectar and pollen availability of each patch type is solely for the creation of input files for BEEHAVE, see Section “Linking BEESCOUT and BEEHAVE”).
3) A single hive, which is defined by its x-y coordinates. This is the location where bees begin and end their scouting trips.
4) A certain number of bees (individual agents), which can either represent honeybees or bumble bees. Bees explore the landscape and detect a flower patch when they enter a grid cell belonging to this patch for the first time of a scouting trip. The *flightPhase* describes the flight pattern of a bee. Phase 1: a bee is leaving the colony in a straight “vector flight” (Riley et al., 2003) towards a field destination (e.g. a flower patch already visited); phase 2: a bee is searching the landscape around its field destination in a small-scale flight pattern; phase 3: a bee is returning in a vector flight to the colony (Fig. 1).

The bees move forward with a constant step length (mean flight speed honeybees: 2.96 m/s (Wright, 2013), bumblebees: 3.22 m/s (Osborne et al., 2013); Supplementary material S5). During *flightPhase* 2, the turning angles are randomly drawn from a distribution, derived from empirical turning angles of searching honeybees or bumble bees (Supplementary material S4). Additionally, bees perform loops (in *flightPhase* 2 only), with the probability *TurnToDestinationProb*, in which case they return linearly to their previous destination and then switch back to the search flight pattern. All flower patches ever detected plus those detected during the current scouting trip are recorded by the individual bee. Flower patches can be detected in any phase, not only in the search phase. Each bee can detect a flower patch only once per scouting trip.

5) A variable number of obstacles in the landscape, representing “lakes” (optionally). Lakes are areas in the landscape that cannot be crossed by bees when they are in the search mode (*flightPhase* 2). However, lakes can be crossed when bees have a destination (e.g. when returning to the hive). This is in accordance with the behaviour of real bees, who are hesitant to fly over water but can cross lakes to collect food (Heran and Lindauer, 1963, von Frisch, 1967, Pahl et al., 2011).

Each time step (“tick”) represents 3 s of real time (i.e. the gap between 2 recordings of a radar-tracked bee). The activities of bees are structured in scouting trips. A scouting trip starts with the bee leaving the hive and heads towards its destination (if any) (determined by the search mode—see below). When the time allowed for a scouting trip is over, the bee returns to the hive and then begins a new scouting trip. The time when a bee returns to the hive is either defined by *TripDuration_s* seconds for all bees or determined randomly for each individual, with an average of *TripDuration_s* seconds. The total duration of the simulation is either defined by a period of *ScoutingPeriod_hrs* hours or by a maximal number of scouting trips per bee, whichever is shorter. Default value of *ScoutingPeriod_hrs* represents an estimation of a bees' total time spent on scouting during its life (for details see Supplementary material S2).

During the setup of the program, parameters are set, the forage map is imported, and the borders of the world are set. The forage map is an image file (supported formats: BMP, JPG, GIF, and PNG) where crop or habitat fields are represented as coloured areas (red, yellow, green, or blue—defined by the user) on an e.g. grey background. These image files may be produced using GIS or graphics software for implementing real maps, or can be created within the BEESCOUT software tool itself for artificial maps. Based on two reference points on the map and the real distance set by the user, the scaling of the map is determined. Flower patches are defined by identifying neighbouring gridcells of identical colour (for details see Supplementary material S2: Sections 5.1. and 5.2), with very large crop or habitat fields being sub-divided in two or more adjoining flower patches, depending on the user's setting of the maximal flower patch size. Optionally, the user can also set crop type, flowering period, nectar and pollen production per m^2^ during flowering period, sugar concentration of the nectar and the bees' handling times for each patch-defining colour (these specifications are only required when creating an input file for the BEEHAVE honeybee model but do not affect the BEESCOUT simulations themselves). Then the landscape is analysed, i.e. the number of flower patches, their size and distance to the hive is determined and bees are created.

In the actual simulation run, bees explore the landscape. At the beginning of their very first scouting trip, bees are naive without any knowledge of the landscape, hence, they have no destination and immediately switch to the search phase (*flightPhase* 2). At the beginning of the second and all following scouting rounds, bees may have a destination, i.e. a location they are heading to. Bees can linearly fly to a flower patch that they, or, depending on the search mode, any other bee in the simulation has been to before. The simulation stops, when either all bees have ceased scouting or the total time allowed for scouting (*ScoutingPeriod_hrs*) is exceeded. Then the detection probabilities for the flower patches are calculated and the results can be saved in an output file, which can serve as an input file for the BEEHAVE colony model (Becher et al., 2014).

### 2.2 Search modes

To define where bees start searching in the landscape after they have left the colony, (described below as the “field destination” i.e. when *flightPhase2* begins) the user can choose between *SearchModes* options. These options take into account possible memory/knowledge of patches already encountered either by the individual bee, or by another bee in the colony. There is little empirical data available to determine how bees make these choices in the field (Biesmeijer and Seeley, 2005, Grüter and Farina, 2009), so different options have been given in the BEESCOUT model to enable theoretical predictions and comparisons.

*SearchModes* options are:

(i) “colony”: no field destination is chosen. On leaving the colony the bee immediately switches to *flightPhase* 2 to search the area around the hive or colony. This would be a typical choice if the bees have not flown in the landscape before, and do not have information from other bees in the colony.
(ii) “known flowerpatch (individual)”: the bee randomly chooses one location that is part of a flower patch already detected by this individual bee. Hence, bigger flower patches are proportionally more likely to be chosen. This could represent the search pattern of bumble bees, where individual bees learn about the landscape, but do not communicate food source locations to their nestmates.
(iii) “known flowerpatch (recruitment)”: similar to “known flowerpatch (individual)”, but now, a location in any patch that was detected by any bee from the colony can become the new destination. Again, the probability of a flower patch being chosen is proportional to its size. This might occur if bees have information from other nestmates about patch locations (from decoding waggle dances for example).
(iv) “furthest location (individual)”: the bee chooses the location which is the furthest from the colony that it has ever been as its new destination. This could represent the search patterns of bees in a poor landscape, with a low density of flower patches, which forces bees to venture further afield.
(v) “visited location (recruitment)”: the new destination of a bee is randomly chosen from a location that was previously crossed by any bee and is not placed in a lake. (Note that bees can cross lakes when they have a destination). This could be a search mode for honeybees in a landscape with highly transient food sources or if the precision of dances is very low (Towne and Gould, 1988, Weidenmüller and Seeley, 1999).
(vi) “last location (individual)”: the last location a bee has been to before it returned to the hive is chosen as the destination. This search mode could apply to honeybees and bumblebees with scouts resuming searching at the previously visited location.
(vii) “mixed strategy (individual)”: before each scouting trip, one of the above *SearchModes* that does not require communication (“colony”, “known flowerpatch (individual)”, “furthest location (individual)”, “last location (individual)”) is randomly chosen.
(viii) “mixed strategy (recruitment)”: before each scouting trip, one of the above colony *SearchModes* (“colony”, “visited NLpatch (recruitment)”, “known flowerpatch (recruitment)”, “furthest location (individual)” “last location (individual)”) is randomly chosen, irrespective of whether or not communication is required. The “mixed strategy” search modes take into account that bees will adapt their search behaviour depending on landscape structure and scouting success.
(ix) “random location”: any location randomly chosen within the maximal foraging range (*MaxForagingRange_m*) of the bees, irrespective of whether it has been visited before. This might reflect a situation of an established honeybee colony in a dynamic landscape, where the colony has explored the complete potential foraging area and food sources could regularly appear and disappear in the landscape. It is not included into the “mixed strategy” search modes as it would overpower the effect of the other search modes.

### 2.3 Linking BEESCOUT and BEEHAVE

BEESCOUT can also be used to create an input file (*NameOutfile*) for BEEHAVE, an agent-based honeybee model that simulates colony development depending on the bees' nectar and pollen foraging activities. This input file contains information on size, location, nectar and pollen availability and detection probabilities of each identified flower patch.

While the detection probabilities and the spatial components (area, distance to the colony etc.) are determined by BEESCOUT, it is up to the user to enter values defining the food availability for each crop or habitat type (though suggested values for some example crop types are provided, see also Supplementary material S8). The input required by the user to create BEEHAVE input files is: the start and stop date of the flowering period, the daily amount of nectar [ml/m^2^] and pollen [g/m^2^] provided, the sugar concentration of the nectar [mol/l] and the handling times [s] to collect a full load of nectar and pollen for each of the up to four different crop or habitat types. Based on the area of each flower patch, the total amount on nectar and pollen provided on each day during the flowering period is then calculated. These data are saved in a text file describing the food availability of each flower patch for each day of the year, hence, the file consists of N flower patches x 365 + 1 (header) lines. The created input file can be read in by BEEHAVE to simulate foraging and colony development in this particular landscape. BEEHAVE in turn offers the option to write the nectar and pollen forager visits of all patches for each day of a one year simulation run into a file (default name: “Input_1-2_Foraging.txt”). This file can then be uploaded by BEESCOUT (interface: “Show foraging data”) to display the nectar and/or pollen visits on the map for each day of a year on a step-by-step basis or as a slide show.

### 2.4 Simulations of detection probabilities

We ran three simulations to (1) validate the model output against empirical data of bumblebee searching flight patterns; (2) compare how honeybees detect flower patches in a landscape when scouting under different *SearchModes* related to the level of communication within the colony; (3) compare detection probabilities of patches that are either unconnected or connected via a linear feature containing forage (representative of a hedgerow) under a search mode involving memory of patch location.

## 3 Results

### 3.1 Simulation 1: comparison of empirical and modelled detection probabilities for bumble bees

#### 3.1.1 Setting

The experiments of Osborne et al. (2013) (which provide the only empirical evidence of naïve bumblebee patch detection) were mimicked in a simulation, to test whether modelled detection probabilities correlate with empirical ones for naïve bumblebees on their first flights from the colony (Model settings: *N_Bees* = 14, *MaxTrips* = 1, *BeeSpecies*: “Bumblebees”, *SearchMode*: “known flowerpatch (individual)”, N_Replicates_ = 30). These were compared against empirical detection probabilities derived from flight tracks of the 14 bumble bees tracked on their first trip (provided in Appendix of Osborne et al., 2013).

#### 3.1.2 Output

**Fig. 2:**
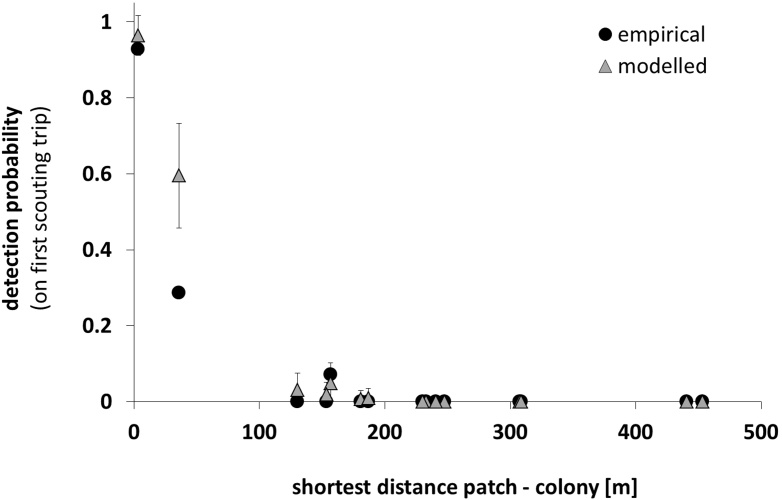
Comparison of empirical and modelled (mean ± s.d.) detection probabilities for 15 flower patches by 14 bumblebees, based on Osborne et al. (2013) (*N_Bees* = 14, *MaxTrips* = 1, *BeeSpecies*: “Bumblebees”, *SearchMode*: “known flowerpatch (individual)”, N = 30).

The forage map had 15 flower patches at different distances from the colony, and the empirical and modelled detection probabilities for the 15 patches were highly correlated (Pearson correlation coefficient, r = 0.96, p < 0.01, N = 15). The relationship between detection probability and distance of the patch from the colony for both empirical and modelled bees are shown in Fig. 2. The model predicts reasonably well whether or not a patch can be found, but there remains some uncertainty about the actual detection probabilities, particularly for patches with intermediate distances.

We also compared flight patterns for bees on later trips when they had more experience of the landscape. Empirical (on first, 2nd or 3rd trip) and modelled (on first and 3rd trip) tracks are shown in Supplementary material S9 and suggest that real bees were more directed in their flights – likely as some of them began to forage and stopped searching whilst the model shows continued searching behaviour – but the flight ranges of modelled and empirical data match well.

### 3.2 Simulation 2: impact of contrasting bee search modes on patch detection

#### 3.2.1 Setting

We compared the output of BEESCOUT in terms of patch detection probabilities for different modes of bee searching to illustrate how it can be used to compare differing bee exploration hypotheses. We used a forage map derived from a realistic map of crops in Hertfordshire in 2009 as the input file to define a realistic landscape (map provided in Supplementary material S6, “RealLandscape.jpg”), with red, blue and yellow grid cells interpreted as mass-flowering patches – or potential sources of nectar and pollen. In this case, the map was simply used for simulating detection of patches at different distances, so it is not important here that in reality there may have been forage in other habitats which is not accounted for by the colour-coding of fields. The hive was placed approximately in the centre of the map. To compare different search strategies we simulated all *SearchModes* options (as listed in Methods). Each simulation (N_Replicates_ = 30) was run with 10,000 honeybees, representing the approximate forager force of a colony (Seeley, 1985). The output generated for each patch (Table 1) was the mean distance to the hive, the area of the patch and the detection probability per trip as well as the total area explored by the bees and the furthest distance to the hive at the end of the run.

#### 3.2.2 Output

**Fig. 3:**
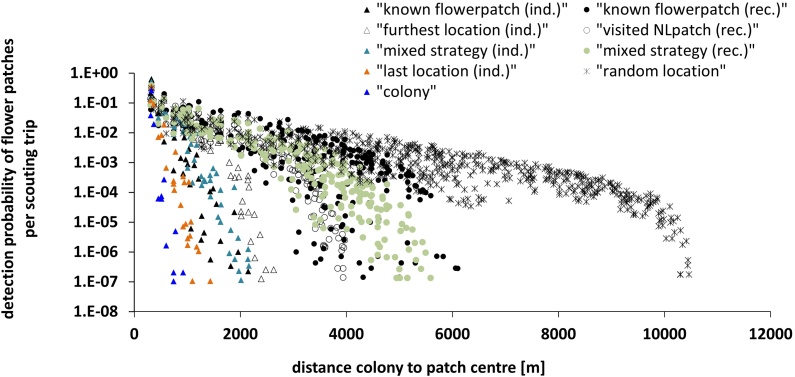
Effect of search modes on detection probabilities (i.e. the likelihood that a bee finds a certain patch during a single scouting trip) of food patches, depending on the distance of the patches. Modelled bee scouts explore the landscape by linearly flying to a field destination and then switch to a smaller scaled search flight. Choice of the field destination depends on the search mode (dimensions landscape: 21.5 × 11 km, 584 identified patches, 30 simulation runs per search mode).

584 patches were identified by BEESCOUT with a distance between 310 and 11,535 m (mean ± s.d.: 5925 ± 2668 m) and an area between 5354 and 422,963 m^2^ (103,192 ± 91,947 m^2^). The search mode “colony” resulted in the smallest area explored by the bees and hence the fewest patches detected (Table 1). The reduction in detection probability with increasing distances was steeper in search modes without communication (“individual”) than with communication (“recruitment”); the widest range of explored area was achieved with the “random location” search mode (Fig. 3).

### 3.3 Simulation 3: connectivity of patches

#### 3.3.1 Setting

To examine whether connectivity in the landscape affects detection of the large distant flower patches, we set up simulations with a large flower patch (radius 120 m, distance 1500 m) that was either connected or not connected to the colony via a narrow strip of flower patches (Supplementary material S6, “Un-/ConnectedPatch.png”). 10,000 honeybee scouts explore the landscape with *SearchMode* set to “known flowerpatch (recruitment)” (N = 30 simulation runs).

#### 3.3.2 Output

**Fig. 4:**
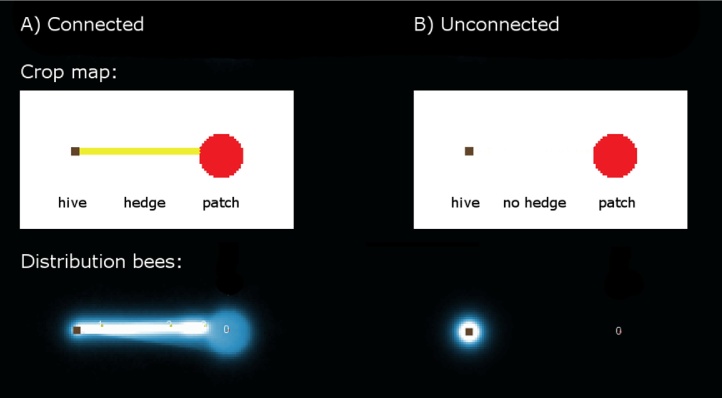
Typical distribution of 10000 bees over 9 h scouting in a landscape with a large flower patch (red) in 1500 m distance that either is (A) or is not (B) connected via a narrow strip of flower patches (yellow) to the colony (brown square) (the white inserts show the actual forage maps loaded into the model). The black to white gradient reflects the number of bees on each grid cell, summed up at each time step over the duration of the simulation run. When scout bees start their new search trips from a flower patch already detected (*SearchMode*: “known flowerpatch (recruitment)”), they are able to find the distant red patch, if it is connected. (For interpretation of the references to colour in this figure legend, the reader is referred to the web version of this article.)

If the large flower patch is connected to the colony then it is detected in each single run with a high detection probability per trip (0.20 ± 0.00), the total area explored by the bees is 2.11 ± 0.03 km^2^ and the furthest distance from the colony is 2068 ± 41 m. If the large patch is not connected via flower strips, it remains undetected in all 30 simulation runs, the total area explored is then only 0.86 ± 0.03 km^2^ and the furthest distance from the colony is 668 ± 67 m. Fig. 4 shows the typical cumulative distribution of the scouts at the end of a simulation run.

## 4 Discussion

BEESCOUT is a model to simulate bee search patterns in any mapped landscape, with defined flower patches or crops. It has flexible settings to enable users to define the search mode of the bees with respect to spatial information and memories available to individual bees and to the colony. Although the harmonic radar technique allows us to accurately record the tracks of flying bees within a range of ca. 1000 m, and we now have information on search patterns under particular conditions for honeybees and bumblebees (Capaldi et al., 2000, Reynolds et al., 2007a, Reynolds et al., 2007b, Reynolds, 2009, Reynolds et al., 2009, Osborne et al., 2013, Degen et al., 2015), it is as yet undetermined as to how most bees explore large landscapes for food. Consequently the probability of finding different patches is still unknown. The model seems to capture detection probabilities for patches close to the colony reasonably well (Fig. 2), but for patches further away, there is a very high degree of uncertainty, mainly because experimental data for large scale search patterns are missing. The variety of search modes in the model means that simulations can explore scenarios of the different types of knowledge that a bee may have about the landscape (Fig. 3 and Supplementary material S10).

Our model may help to inversely narrow down the possible range of search modes of real bees by comparing model outputs to the sparse information available, for example about maximal flight distances. Experiments suggest that bees fly to a known food source and, if it is no longer present, search the vicinity (Reynolds et al., 2009). This behaviour is represented in the search modes “known flowerpatch (colony/individual)". However, the emergent maximal range for honeybees using this search mode in the model is ca. 6 km (Table 1), which is still below the foraging ranges of real honeybees which can forage for distances over 10 km from the hive (Beekman and Ratnieks, 2000).

This difference might be explained by the ability of a colony to inherit knowledge of patch locations from the previous worker generation via waggle dances (Biesmeijer and Seeley, 2005), whereas our simulations represent the situation of a first, naive worker generation placed into a new environment. To model the situation of an established colony in a well-known area, where experienced foragers have already gained knowledge of patches in large distances, the search mode “random location” might be more suitable. Here, even patches far away from the hive can be found as long as they are within the maximal foraging range of the bees. In this case, the detection probability should rather be calculated from the distance (Table 1) then simulated by the scouting model.

Landscape structures like roads or field margins can guide bees in reality (Dyer, 1996, Wolf et al., 2014), whether or not they contain forage. In the model this is only reflected by the connectivity of floral patches, but it can have a huge effect on the virtual bees’ flight paths (Fig. 4). Further simulations are required to determine how the spatial contiguity of patches affects detection rate of flower patches over time and space.

There are some important constraints to the model. A “detection” of a patch only takes place when a bee enters the actual area of a flower patch. In reality, bees will perceive a flower patch based on visual and/or olfactory cues before they reach it. An object can be seen by a honeybee, when the visual angle is greater than ca. 5° (Giurfa et al., 1996). As flight heights of bees are usually low (Riley et al., 1999), the visual angle of even large fields is relatively small (assuming a flat horizontal topography) so that the visual detection range is only marginally larger than the field itself (using trigonometry, one can calculate that e.g. a field with a diameter of 500 m can be seen by a bee flying 1.9 m high (Riley et al., 2005) from a distance of no more than 21 m). Hence, by not including visual perception range in the model we only slightly underestimate the detection probabilities.

Far more difficult to assess is the impact of olfaction, as direction and intensity of a floral scent plume will vary for different plant species and especially with wind conditions (Wright and Schiestl, 2009). Air turbulence will make it difficult for the bees to follow an odour “uphill” to the source (Reinhard and Srinivasan, 2009) and an upwind zig-zag flight within the scent plume, as suggested by Wenner et al. (1991), is likely to be prohibitively time- and energy-consuming for long-distance search flights (Reinhard and Srinivasan, 2009) and has to our knowledge not been observed in reality (Reynolds et al., 2009). Given the current uncertainties around how bees respond in different wind conditions, we have not modelled this process, but when clear empirical information becomes available, the model can be modified accordingly. Thus the current model probably underestimates the detection probabilities in most cases, particularly for close flower patches.

It should also be noted that the detection probability is not necessarily a predictor for the actual exploitation of a patch, as bees prefer food sources with higher sugar concentrations, or higher pollen volume, and closer to the colony (for energetic reasons). Hence, patches far away from the colony, where the uncertainty for the detection probability is highest, will in most cases be the ones with the least effect on colony survival. A honeybee colony will collect the majority of its nectar and pollen from food sources nearer than 2 km (Visscher and Seeley, 1982, Steffan-Dewenter and Kuhn, 2003), although foraging distance can vary with season (Beekman and Ratnieks, 2000, Couvillon et al., 2014).

Because we still do not know exactly how bees explore a landscape, BEESCOUT is a useful tool for learning and making relative predictions rather than for making absolute predictions. It can also guide the collection of new data and the design of new experiments. BEESCOUT has been implemented in NetLogo (Wilensky, 1999) precisely because this software is free and easy to learn and modify, so it is useable by anyone wishing to investigate the relationship of Central Place Foragers with their landscape. BEESCOUT helps elucidate how landscape configurations and parameters describing searching behaviour affect detection probabilities and thus, ultimately, bee colony health and pollination services. More broadly, the looping search patterns performed by bees are also detected in other organisms (e.g. in butterflies: Cant et al., 2005, Heinz et al., 2007) and this type of software tool could be modified to explore how other animals might find resources, such a seed eating birds or seabirds. By using BEESCOUT for a range of real and hypothetical, stylized landscapes, the user will learn to ask targeted questions, create specific hypotheses, perform key experiments and collect more relevant data that will allow the investigator to inversely determine, e.g., the “optimal” or actual search modes used by a given species in a certain landscape.

BEESCOUT is also a software tool to implement seasonal nectar and pollen availability in realistic landscapes. It can store detailed information provided by the user about beginning and end of flowering periods, crop type, and nectar and pollen provisioning of the different flower patches. This information can then be exported into the BEEHAVE model for the prediction of the overall impact of multiple stressors on bee colonies. We have thus implemented the EFSA (2015) recommendation that the representation of the landscape in BEEHAVE should be extended. BEESCOUT is thus a robust and flexible tool for scientists and practitioners to start exploring how individual bee scouting behaviour might affect a bee colony’s response to changing landscapes and forage availability.

## Figures and Tables

**Table 1:** Overview of search modes. *Area* refers to the area explored by the bees, based on the number of grid cells visited by at least one bee. *Max. displacement* is the maximal distance ever reached during a simulation run between a bee and the colony. *Patches detected* refers to the number of food patches discovered by the bees during a simulation run. *Lambda* is the coefficient of an exponential fit (detection probability = e^λ*distance^) with values closer to zero resulting in a larger area explored (Fig. 3).

Search mode	Area [km^2^] (mean ± sd)	Max. displacement [m] (mean ± sd)	# patches detected	Lambda
“colony”	1.0 ± 0.0	681 ± 54	12	−0.02318
“last location (individual)"	2.9 ± 0.1	1137 ± 70	26	−0.01347
“known flowerpatch (individual)"	4.7 ± 0.2	1982 ± 165	36	−0.00783
“mixed strategy (individual)"	8.5 ± 0.2	2101 ± 117	49	−0.00740
“furthest location (individual)"	14.3 ± 0.1	2350 ± 57	59	−0.00524
“visited NLpatch (recruitment)"	36.8 ± 0.4	3729 ± 64	137	−0.00282
“known flowerpatch (recruitment)"	42.0 ± 2.5	5799 ± 147	213	−0.00156
“mixed strategy (recruitment)"	47.5 ± 1.4	5377 ± 169	219	−0.00214
“random location”	208.3 ± 0.3	10,207 ± 47	560	−0.00073

## Appendix A Supplementary data

S1: Implementation of the BEESCOUT model in NetLog.

S2: ODD protocol of the model.

S3: A user's manual for the BEESCOUT software tool.

S4: Excel tables listing the scheduling, procedures, global variables, entities state variables, display buttons, default setting and histograms of turning angles.

S5: Methods harmonic radar experiments.

S6(a): Input maps used for the simulations in this publication.

S6(b): Input maps used for the simulations in this publication.

S6(c): Input maps used for the simulations in this publication.

S6(d): Input maps used for the simulations in this publication.

S7: Copyright & GNU General Public License of BEESCOUT.

S8: Tables on nectar and pollen production of some crop species.

S9: Comparison of modelled and empirical bumblebee flight tracks.

S10: Sensitivity analysis.
