# Supplementary material for: BEESCOUT: A model of bee scouting behaviour and a software tool for characterizing nectar/pollen landscapes for BEEHAVE
Source: Ecol Modell. 2016 Nov 24;340:126–33. doi: 10.1016/j.ecolmodel.2016.09.013 (PMC5070411; doi:10.1016/j.ecolmodel.2016.09.013)
Supplement: Supplementary file 2 — S2: ODD protocol of the model. [file mmc2.pdf]

BEESCOUT: a model of bee scouting behaviour and a software tool for characterizing nectar and pollen landscapes for BEEHAVE

Supplementary material to:

> Becher, M. A., Grimm, V., Knapp, J., Horn, J., Twiston-Davies, G., Osborne, J.L. (2016) BEESCOUT: a model of bee scouting behaviour and a software tool for characterizing nectar/pollen landscapes for BEEHAVE. *Ecological Modelling*.

BEESCOUT is computer program implemented in NetLogo (Wilensky 1999) which includes four modules which are all based on the same kind of representation of a landscape in which honeybees or bumblebees can forage. The first two modules are applicable to both honeybees and bumblebees, the other two modules are related to the honeybee colony model BEEHAVE:

I. The model BEESCOUT which simulates scouting of honeybees and bumblebees in given landscape. The landscape can be imported from input files (module II).
II. A module for creating and modifying a landscape of flowering patches from input files.
III. A module for defining food flow and creating corresponding input files for the BEEHAVE colony model.
IV. A module for visualising foraging activities in a given landscape, using output from the BEEHAVE colony model.

For information about how to actually run and use the NetLogo program, see the User Manual.

I. The foraging model BEESCOUT 2
1. PURPOSE 2
2. ENTITIES, STATE VARIABLES, AND SCALES 2
3. PROCESS OVERVIEW AND SCHEDULING 8
4. DESIGN CONCEPTS 12
5. INITIALISATION 13
5.1 Setup and importing a crop map 13
5.2. Analysis of the landscape and determination of flower patches 14
6. INPUT DATA 15
7. SUBMODELS 16
II. A module for creating and modifying a landscape of flowering patches. 22
III. Define food flow and create inputfile for BEEHAVE colony model 23
IV. Display of foraging activities, modelled in BEEHAVE 24
REFERENCES 24

I. The foraging model BEESCOUT

The model description follows the ODD (Overview, Design concepts, Details) protocol, a standard format for describing individual-based models (Grimm et al. 2006, 2010). The model was implemented in NetLogo (Wilensky, 1999), version 5.0.1.

1. PURPOSE

The main purpose of the landscape model is to determine the number, location and size of food sources for a honey bee colony on the basis of real or artificial crop maps and to assess the probabilities of these food sources to be detected by a scouting bee. Alternatively, also detection probabilities for bumblebees can be explored.

2. ENTITIES, STATE VARIABLES, AND SCALES

The model includes the following entities:

1) Grid cells constituting the landscape in which bees can search for food sources ("flower patches"). The landscape is a forage map specifying patches where nectar and/or pollen is available in flowers. The map is either imported by the user as an image file, or one can be generated within the program (BEESCOUT modules II and III). State variables of grid cells are listed in Table 1.

2) A certain number of flower patches, which represent fields with crops or wild flowers, as defined by the user. A patch is defined if there is a contiguous area of Netlogo grid cells of one colour on the map. It is possible to define four different patch types using four colours (red, green, yellow and blue). The state variables of flower patches are listed in Table 2.

3) Optionally a variable number of obstacles in the landscape, representing 'lakes'. Lakes are areas in the landscape that cannot be crossed by bees when they are in a specific search mode (*flightPhase* 2; see below). However, lakes can be crossed when bees have a destination (e.g. when returning to the colony). This is in accordance with the behaviour of real bees, who are hesitant to fly over water but can cross lakes to collect food (Heran & Lindauer 1963, von Frisch 1967, Pahl et al. 2011).

4) A single colony, which is defined by its x-y coordinates. This is the location where bees begin and end their scouting trips.

5) Bees (individual agents), which can either represent honeybees or bumblebees. The bees' state variables are listed in Table 3.

   Bees are also characterized by the "search mode". Since this mode in most simulations is identical for all bees, it has not been implemented as a state variable of bees but as a simulation parameter: to define where bees start searching in the landscape after they have left the colony, (described below as the 'field destination' i.e. when *flightPhase2* begins) the user can choose between *SearchModes* options. These options take into account possible memory/knowledge of patches already encountered either by the individual bee, or by another bee in the colony. There is little empirical data available to determine how bees make these choices in the field (Biesmeijer & Seeley 2005, Grüter & Farina 2009), so different options have been given in the model to enable theoretical predictions and comparisons. Since in a given simulation all bees have the same search mode, search mode is not a state variable of the bees but of the entire model world.

   SearchModes options are:

   (i) "colony": no field destination is chosen. On leaving the colony the bee immediately switches to *flightPhase* 2 to search the area around the colony. This would be a typical choice if the bees have not flown in the landscape before, and do not have information from other bees in the colony.

   (ii) "known flowerpatch (individual)": the bee randomly chooses one location that is part of a flower patch already detected by this individual bee. Hence, bigger flower patches are proportionally more likely to be chosen. This could represent the search pattern of bumblebees, where individual bees learn about the landscape, but do not communicate food source locations to their nestmates.

   (iii) "known flowerpatch (recruitment)": similar to "known flowerpatch (individual)", but now, a location in any patch that was detected by any bee can become the new destination. Again, the probability of a flower patch to be chosen is proportional to

its size. This might occur if bees have information from other nestmates about patch locations (from decoding waggle dances for example).

(iv) "furthest location (individual)": the bee chooses the location which is the furthest from the colony that it has ever been as its new destination. This could represent the search patterns of bees in a poor landscape, with a low density of flower patches, which forces bees to venture further afield.

(v) "visited location (recruitment)": the new destination of a bee is randomly chosen from a location that was previously visited by any bee and is not placed in a lake. (Note that bees can cross lakes when they have a destination). This could be a search mode for honeybees in a landscape with highly transient food sources or if the precision of dances is very low (Towne & Gould 1988, Weidenmüller & Seeley 1999).

(vi) "last location (individual)": the last location a bee has been at before it returned to the colony is chosen as destination. This search mode could apply to honeybees and bumblebees with scouts resuming searching at the previously visited location.

(vii) "mixed strategy (individual)": before each scouting trip, one of the above *SearchModes* that do not require communication ("colony", "known flowerpatch (individual)", "furthest location (individual)", "last location (individual)") is randomly chosen.

(viii) "mixed strategy (recruitment)": before each scouting trip, one of the above colony *SearchModes* ("colony", "visited NLpatch (recruitment)", "known flowerpatch (recruitment)", "furthest location (individual)" "last location (individual)") is randomly chosen, irrespective if or if not communication is required. The "mixed strategy" search modes take into account that bees will adapt their search behaviour depending on landscape structure and scouting success.

(ix) "random location": any location randomly chosen within the maximal foraging range (*MaxForagingRange_m*) of the bees, irrespective of whether it has been visited before. This might reflect a situation of an established honeybee colony in a dynamic landscape, where the colony has explored the complete potential foraging area and food sources could regularly appear and disappear in the landscape. It is not included into the "mixed strategy" search modes as it would overpower the effect of the other search modes.

The model world consists of 300 x 210 grid cells. The size of the area represented by the model world depends on the scaling of the forage map. Scaling is incorporated into the model by the user, relating the distance between two grid cells in the modelled world to the real distance represented. The model world has closed boundaries, i.e. if bees reach the borders of the world, they bounce back by randomly changing their direction.

Time is represented via discrete time steps, or “ticks”. Each time step represents *Gap_s* [s] of real time (default: 3s). The activities of bees are structured in scouting trips. A scouting trip

starts with the bee leaving the colony and heading towards her destination (if any) (determined by the search mode – see below). When the time allowed for a scouting trip is over, the bee returns to the colony and then begins a new scouting trip. The duration of a scouting trip is either set to *TripDuration_s* seconds for all bees or determined randomly for each individual, with an average trip duration of *TripDuration_s* seconds. The total duration of the simulation is either defined by a period of *ScoutingPeriod_hrs* hours or by a maximal number of scouting trips per bee, whichever is shorter. Default value of *ScoutingPeriod_hrs* represents an estimation of a bees' total time spent on scouting during its life.

Table 1: State variables of grid cells (Netlogo *patches*):

Variable	Comment
firstPatchOfFlowerpatch	a grid cell (NetLogo 'patch') that is defined as food source (red, green, yellow or blue) but has not yet been assigned to a flower patch in AnalyseProc. It then becomes the first cell of a newly created flower patch.
flowerPatchID	the ID of a flower patch (initial value: -1; final value: 0..N patches)
mapDisplay	represents the value displayed when pressing the buttons "Detection Prob" or "History"
originalColor	the color of the grid cell after the InputFile was imported (ImportMapProc); can be displayed by pressing the button "Original color"
patchColor	the main color of a grid cell: red, green, yellow, blue for potential food sources, light blue for lakes and obstacles, grey for the matrix and magenta for the borders of the world
satelliteColor	the color of the grid cell after the SatelliteFile was imported (Setup); can be displayed by pressing the button "Satellite"
visits	the total number of times a grid cell is crossed by a bee

Table 2: State variables of *patchStatistics*, storing the data of flower patches.

Variable	comment
areaPx	number of grid cells belonging to a certain flower patch
areaSqm	area [m^2] of a flower patch
calculatedDetectionProb_per_trip	probability that a flower patch is detected by an individual bee during a single scouting trip, calculated from its size and distance to the colony
closestDistance_m	the shortest distance [m] between a flower patch and the

	colony
concentration	sugar concentration [mol/l] of the nectar provided at a flower patch; only used to create an input file for the BEEHAVE model
detects1000List	a list, saving the current total number of detections of a flower patch every 1000 time steps
firstDetection_s	time [s] until the first detection of a flower patch
maxDetectProb_per_s	the highest detection probability (per s) that was ever reached for a flower patch
meanDistance_m	distance [m] between the patch centre and the colony
modelledDetectionProb_per_s	probability that a flower patch is detected by an individual bee per second of scouting, based on the results of the simulation
modelledDetectionProb_per_trip	probability that a flower patch is detected by an individual bee during a single scouting trip, based on the results of the simulation
nectarGathering_s	a bees' handling time [s] at a patch to collect a full nectar load; only used to create an input file for the BEEHAVE model
nNectarVisits	number of times a patch has been visited by nectar foragers on the current day as a result from a BEEHAVE simulation run (data imported from InputputForagingFile)
nPollenVisits	number of times a patch has been visited by pollen foragers on the current day as a result from a BEEHAVE simulation run (data imported from InputForagingFile)
patchType	type of flower patch (e.g. "Oilseed rape"); only used to create an input file for the BEEHAVE model
pollenGathering_s	a bees' handling time [s] at a patch to collect a full pollen load; only used to create an input file for the BEEHAVE model
quantityNectar_l	total amount of nectar [l] available at a flower patch during flowering period; only used to create an input file for the BEEHAVE model
quantityPollen_g	total amount of pollen [g] available at a flower patch during flowering period; only used to create an input file for the BEEHAVE model

startDay	first day of a flower patch's flowering period
stopDay	last day of a flower patch's flowering period
timeMaxDetectProb_s	time [s] when the highest detection probability (per s) ever was reached in a flower patch
totalDetections	total number of detections of a flower patch
visitColor	color to represent the number of nectar, pollen or total visits at a flower patch (data from InputForagingFile)

Table 3: State variables of *bees*.

Variable	comment
destinationList	x-y coordinates of the bees' destination, which can either be a location in the landscape or the location of the colony
flightPhase	flight phases are: phase 1: a bee is leaving the colony in a vector flight towards a field destination; phase 2: a bee is searching the landscape around its field destination in a small-scaled flight pattern; phase 3: a bee is returning in a vector flight to the colony
furthestDistance_m	saves the furthest distance from the colony an individual bee has ever been
furthestLocationList	saves the x-y coordinates of the furthest location from the colony an individual bee has ever been
knownPatchesList	a list with the ID of all flower patches known to the bee
lastDestinationList	x-y coordinates of the bees' last field destination or the location of the colony, if a bee has never been at a field destination
lastLocationList	the bees' location in the previous time step (only updated while the bee is searching, i.e. in flightPhase 2, when bee is white or orange)
nowDetectedPatchesList	ID of those patches, detected by a bee in the current scouting trip
nTrips	the bees' number of completed scouting trips
time_s	total time passed [s]

3. PROCESS OVERVIEW AND SCHEDULING

In the actual simulation run, bees explore the landscape (procedure *MoveBeesProc*, see Table 4). Bees detect a flower patch when they enter a grid cell belonging to this patch for the first time of a scouting trip. The *flightPhase* describes the flight pattern of a bee:

phase 1: a bee is leaving the colony in a vector flight towards a field destination (i.e. a location in the landscape the bee is trying to reach, choice of this location depends on the search mode - see below);

phase 2: a bee is searching the landscape around its field destination in a small-scaled flight pattern;

phase 3: a bee is returning in a vector flight to the colony.

The bees move forward with a constant step length and the turning angles in *flightPhase* 2 are randomly drawn from a distribution, derived from empirical turning angles of searching honeybees or bumblebees. Additionally, bees perform loops (in *flightPhase* 2 only), with the probability *TurnToDestinationProb*, in which case they return linearly to their previous destination and then switch back to the search flight pattern. All patches ever detected and patches detected during the current scouting trip are recorded by the individual bee. Flower patches can be detected in any phase, not only in the search phase. Each bee can detect a flower patch only once per scouting trip

When they head to a destination or return to the colony, they show a linear flight pattern (*flightPhase* 1 or 3). When they are in the search phase (*flightPhase* 2), they show a smaller scaled flight pattern. At the beginning of their very first scouting trip, bees are naive without any knowledge of the landscape, hence, they have no destination and immediately switch to the search phase. At the beginning of the second and all following scouting rounds, bees may have a destination. Depending on *SearchMode*, bees can linearly head to a flower patch or a location either they or any other bee has been before. The simulation stops, when the total time allowed for scouting (*ScoutingPeriod_hrs*) is exceeded. Then the detection probabilities for the flower patches are calculated and the results can be saved in an output file.

Table 4 provides and overview of all procedures used in the NetLogo program implementing BEESCOUT. Table 5 represents the scheduling of all procedures of all BEESCOUT modules. The scheduling of the scouting model (module I) is marked via yellow background.

Table 4: Overview of all procedures (procedures marked with † link the BEESCOUT model to the BEEHAVE colony model)

Procedure	Description
AnalyseOutfileProc	Setup and run of the model to determine the detection probabilities, results are written in an outfile for the BEEHAVE model
AnalyseProc	determination of flower patches, calls procedures to create scout bees, and exploration of landscape
ClearBeesProc	kills existing and creates new bees, plots etc. are cleared, detection probabilities are set to 0, random-seed set to initial value
CreateBeesProc	creates scout bees
CreatePatchStatisticsProc	creates patchStatistics for each flower patch, to store the flower patch data
Default_ButtonProc	sets all parameters on the interface to their default values
DetectionProc	determines if/which flower patch is detected by a bee
DetermineBordersProc	determines the bordes of the map which cannot be crossed by the bees
DetermineSizeProc	determines the size and distance to colony of the flower patches
DoDetectionPlotsProc	updates the plot "detection probability per trip"
Go	runs the individual-based scout model to determine the detection probabilities of the flower patches
ImportMapProc	imports an image file (forage map) and corrects colours
MoveBeesProc	the movement of the bees
ReadForagingDataProc †	reads in the number of nectar and pollen visits for each patch from the InputForagingFile, which was created during a previous simulation run of the BEEHAVE model
Setup	clears all variables, resets time, sets initial conditions, creates colony
ShowForagingDataProc †	displays nectar or pollen visits for all flower patches on the map, based on a BEEHAVE simulation
TurnDestinationProc	new direction of the bee if it has a destination
TurnNoDestinationProc	new direction of the bee if it has no destination
WriteFromListToFileProc †	creates an input file for the BEEHAVE model

Table 5: Scheduling of the procedures. Level 1 procedures are called by buttons on the interface, procedures in level 2 and 3 are called by the procedure of the next higher level.

**Level 1**	**Level 2**	**Level 3**	**Condition**
Setup			Button: "Setup"
	ImportMapProc		if InputFile != "No input file"
	DetermineBordersProc		if InputFile != "No input file"<br>AND count patches with [originalColor = BorderColor] > 0
	AnalyseProc		
		CreatePatchStatisticsProc	
		CreateBeesProc	
		DetermineSizeProc	
Go			Buttons: "Step", "Go"; repeated once or continuously
	MoveBeesProc		
		TurnNoDestinationProc	if destinationList is empty
		TurnDestinationProc	if destinationList is not empty
		DetectionProc	
	DoDetectionPlotsProc		if count PatchStatistics > 0
ReadForagingDataProc			Button: "Read foraging data"
ShowForagingDataProc			Buttons: "Read foraging data", "Go to day 1", "Refresh",<br>"Slide show", "-30d".."+30d"

AnalyseOutfileProc			Button: "Analyse & Outfile"
	Setup		
	Go		repeated for ScoutingPeriod_hrs * 3600 / Gap_s
	WriteFromListToFileProc		
ClearBeesProc			Button: "Clear Bees"
Default_ButtonProc			Button: "Default (Honeybees)"
BumblebeesProc			Button: "Bumblebees"
HoneybeesProc			Button: "Honeybees"

4. DESIGN CONCEPTS

Basic principles

The model simulates only scouting trips of honeybees and bumblebees, i.e. the search for new food sources. It does not simulate the exploitation of known flower patches. Hence, there is no foraging for nectar or pollen implemented, and patches only differ in their size, shape and location, but do not have any sort of attractiveness (The nectar and pollen flow for the different flower patch types that is defined on the interface is only used to create an input file for the BEEHAVE model. It is *not used* within the BEESCOUT model). There is no mortality, so the number of bees remain constant during a simulation run. The movement of bees during their search phase is determined by a turning angle and the step width, both based on empirical data from radar tracked honeybees amd bumblebees. The turning angle (i.e. the change in direction) is randomly drawn from a distribution, whereas the step width is constant. Choice of the location, where the bee begins its actual search, is based on the idea that bees would visit a known location, may find it unrewarding and then start to search for a new food source from their current location. As honeybees can recruit colony members to profitable food sources by waggle dances, destinations are not necessarily restricted to those locations already visited by the individual bee. For bumblebees, who do not communicate the location of a food source, only information gathered from an individual is used determine the field destination, where the bee switches to the search flight.

Emergence

Detection probabilities of the flower patches emerge from the bees' flight patterns and the landscape structure.

Sensing

Bees sense a flower patch or a lake, when they reach it and they know their own location and the location of their colony. They remember flower patches they have detected on a previous trip, flower patches they have detected on the current trip as well as the last location and the furthest location they have ever been.

Interaction

Bees search for food patches individually, but can interact via information transfer. They can share information of detected food sources via the location of the "new field destination" if the search mode is "recruitment" (e.g. *SearchMode* "known flowerpatch (recruitment)"). This reflects the foraging behaviour of bees with a recruitment system, e.g. honeybees. If the search mode is "individual" they do not share information, which would reflect the foraging behaviour of e.g. bumblebees or aggregations of solitary bees; consequently, there is no interaction among bumblebees.

Stochasticity

The directions of bees' turning angle is stochastically determined from a given distribution. If *RandomTripDuration* is true, then also the duration of a bees' scouting trip is randomly determined

Observation

From the analysis of the imported map, the number of flower patches, their size and their distance to the colony is calculated and displayed on the interface. The number of detections is recorded and used to calculated the modelled detection probability per second and per scouting trip. The time of the first detection of a flower patch as well as the number of bees that crossed a grid cell are recorded. All this information can be displayed on the map of the interface. A plot can be used to illustrate the detection probability over time for up to four patches. The total area explored by the bees and the furthest distance of bee from the colony are also displayed.

5. INITIALISATION

The model is initialised in the procedure *Setup* (*ImportMapProc*, *DetermineBordersProc* and *AnalyseProc* (*CreateStatisticsProc*, *CreateBeesProc*, *DetermineSizeProc*)). In the *Setup* procedure, time and agents are cleared, parameters and the random seed are set to their initial values. Then a forage map (if provided) is imported (*ImportMapProc*) and the borders of the world are set (*DetermineBordersProc*). Colour shades are translated into a limited number of primary colours. Finally, the scale of the map is calculated, using the real distance between two reference points. *AnalyseProc* determines the number of flower patches in the landscape. *PatchStatistics* (i.e. representations of flower patches) are then created in *CreateStatisticsProc* to keep track of the number of detections for each flower patch. In *CreateBeesProc* bees are created and *DetermineSizeProc* determines the area of the flower patches. Then lakes are defined and an output file may be created in *WriteFromListToFileProc*.

5.1 Setup and importing a crop map

In the Setup procedure, some parameters are set to their initial value. A *hive* (colony) is created and placed at the location *Col_X, Col_Y*. Then the colour (*pcolor*) of all grid cells (Netlogo *patches*) is set to the unique *BorderColour*. The list *PatchColoursList* is populated with the colours of patch types (*RedPatches*, *GreenPatches* etc.) included in the analysis. To calculate the correct scaling for the map, the x-coordinates of two reference points *(Scale_X1*, *Scale_X2*) have to be chosen and the real distance [m] between these points has to be provided (*ScaleDistance_m*):

```
set Scaling ScaleDistance_m / (Scale_X2 - Scale_X1)
```

If an input file for a forage map is provided, the file is imported in the procedure *ImportMapProc*. After the file was imported, each grid cell (Netlogo *patch*) saves its colour (*pcolor*) in the variable *originalColor*. Some shades of colours are then converted into the related prime colour (i.e. into red, green, blue, yellow, white or black). The range of colours converted into the prime colour are defined by *Red_min, Red_max* for red (Netlogo colour value: 15), *Yellow_min, Yellow_max* for yellow (colour value: 45), *Green_min, Green_max* for green (colour value: 55), and *Blue_min, Blue_max* for blue (colour value 105). Black is represented in Netlogo by colour values of 0, 10, 20.. 130 and white is represented by 9.9,

19.9, 29.9.. 139.9. Hence, very dark colours with `remainder pcolor 10 < Black_th` are set to black and very light colours with `remainder pcolor 10 > White_th` are set to white. Each grid cell (Netlogo *patch*) then saves its colour in its variable *patchColor* and *flowerPatchID* is set to -1, indicating that the grid cell does not (yet) belong to a flower patch.

Then the borders of the map are determined in the procedure *DetermineBordersProc*. The borders are those areas on the top and bottom or on the left and the right, with *pcolor* = *BorderColor* (i.e. the color they were set to before the map was imported. Almost certainly, grid cells have changed their colour to the value provided by the imported image). The top border, for example, is then defined by the y-coordinate of those patches with the color *BorderColor* in the upper half of the world, which have the lowest y-coordinate:

```
set TopBorder min [ pycor ] of patches with
[(pycor > (max-pycor / 2)) and (originalColor = BorderColor)]
```

The other three borders are determined in a similar way. Borders are important, as they cannot be crossed by the bees.

If a satellite or aerial image is provided, it is imported and each grid cell (Netlogo *patch*) then saves its color in its variable *satelliteColor*.

5.2. Analysis of the landscape and determination of flower patches

Flower patches are determined by a number of neighbouring grid cells of the same colour indicating food sources:

First, some variables are set (procedure: *AnalyseProc*):

```
let currentColor 0

let currentPatchID -1

let flowerPatchCounter 0

set Repetitions round (MaxPatchRadius_m / Scaling)
```

Repetitions affects the maximal size a flower patch can have. If a field is larger, it will be divided in several flower patches.

Then *patchColor* of all grid cells (NetLogo *patches*) is compared in an ordered way (from top left to bottom right corner) to *PatchColoursList*, which contains the up to four colours (red, green, yellow, blue) that define food sources:

```
foreach sort patches [ ask ?
 [
  if member? patchColor PatchColoursList
  [ ...
```

If a grid cell has a *patchColour* defined as food source and it was not assigned to an already existing flower patch yet (i.e. *flowerPatchID* of the grid cell is still set to its initial value of -1), then a new flower patch is being created:

```
    if flowerPatchID = -1
    [
```

```
set flowerPatchID (flowerPatchCounter)
set currentColor pcolor
set firstPatchOfFlowerpatch true
set currentPatchID flowerPatchID
```

FlowerPatchID of the grid cell is set to current value of the flower patch counter (*flowerPatchCounter*), its colour and flowerPatchID are saved in *currentColor* and currentPatchID (to make sure that neighbouring flower patches of different colours are not merged). Then the (eight) neighbouring grid cells are added to the new flower patch, if they have the same colour. This process is then repeated *Repetitions* times, always checking if the neighbours of those grid cells already belonging to this new flower patch have the correct colour and are not assigned to a flower patch yet. After the last repetition, *flowerPatchCounter* is increased by 1:

```
            repeat Repetitions
            [
             ask patches  with [(pcolor = currentColor) and (flowerPatchID
                  = currentPatchID)]
               [
                ask neighbors with [(pcolor = currentColor) and
                                    (flowerPatchID = -1)]
                  [ set flowerPatchID currentPatchID ]
                ]
             ]
            set flowerPatchCounter flowerPatchCounter + 1
         ] ] ] ]
```

The relevant data for each flower patch (like area, number of detections etc.) is saved in the entities *patchStatistics* (Tab. 3). For each flower patch, a *patchStatistic* is created (*CreatePatchStatisticsProc*). Then *bees* are created (*CreateBeesProc*) and and the position of the *hive* (colony) is defined.

If the Netlogo switch *Lakes* is set true, then grid cells with a black *patchColor* are interpreted as water (or other obstacles with a similar effect), and their *patchColor* is reset to *LakeColor*. This will affect the movement of the *bees* (MoveBeesProc) who cannot cross these grid cells while they are in the search mode (flightPhase 2). However, water can be crossed when bees are heading to a field destination or are returning to the colony (Heran & Lindauer 1963, von Frisch 1967, Pahl et al. 2011).

6. INPUT DATA

BEESCOUT does not import data representing time series of driving variables, for example weather.

Optionally, an input file, created by a previous simulation run of the BEEHAVE model with the same landscape can be read in. This input file does not affect the simulation run of the BEESCOUT model but only serves to visualise the foraging activities of bees as they were recorded during a previous run of the BEEHAVE model in the same landscape. The input file contains the number of all visits of nectar and pollen foragers at the flower patches for every

day of one year. The landscape model can then visualise the foraging patterns the bees showed during the BEEHAVE simulation. The input file has the following format:

551

day who nectarVisits pollenVisits

1 0 0 0

1 1 0 0

1 2 0 0

...

365 549 0 0

365 550 0 0

365 551 0 0

The first line shows the number of flower patches, the second line is the header for the four colums: day of the year (1 - 365), the patch ID, the number of recorded nectar visits on that day at the specified patch and the number of recorded pollen visits on that day at the specified patch. E.g. 181 112 100 300 would mean that 100 nectar foragers and 300 pollen foragers had visited patch number 112 on day 181 in the BEEHAVE simulation.

7. SUBMODELS

The scouting efforts of a honeybee colony depends on forage availability in the landscape and ranges from ca. 5% to ca. 35% of all foragers (for "rich" and "poor" forage conditions, Seeley 1983). The average total flight performance of a honey bee is ca. 500 km during its lifetime (Neukirch 1982; J Comp Physiol: 146, 35-40). Therefore, we estimate the total scouting distance of a bee to range from ca. 25 km to 175 km. Assuming an average flight speed of 3 m/s (based on our harmonic radar data), a honey bee spends between ca. 2 hrs to 16 hrs with scouting. We hence set *ScoutingPeriod_hrs* to 9 [hrs] for honeybees and, lacking equivalent data, also for bumblebees.

Perception (i.e. "detections") of flower patches in the model only takes place when *bees* enter the patch. In reality, bees will perceive a flower patch based on visual and/or olfactory cues before they reach it. An object can be seen by a honeybee, when the visual angle is greater than ca. 5° (Giurfa et al. 1996). As flight heights of bees are usually low (Riley et al. 1999), the visual angle of even large fields is relatively small (assuming a flat horizontal topography) so that the visual detection range is only marginally larger than the field itself (using trigonometry, one can calculate that e.g. a field with a diameter of 500 m can be seen by a bee flying 1.9 m high (Riley et al. 2005) from a distance of no more than 21 m). Hence, by not including visual perception range in the model we only slightly underestimate the detection probabilities. Far more difficult to assess is the impact of olfaction, as direction and intensity of a floral scent plume will vary for different plant species and especially with wind conditions (Wright & Schiestl 2009). Air turbulence will make it difficult for the bees to follow an odour "uphill" to the source (Reinhard & Srinivasan 2009) and an upwind zig-zag

flight within the scent plume, as suggested by Wenner et al. (1991), is likely to be prohibitively time and energy consuming for long-distance search flights (Reinhard & Srinivasan 2009) and has never been observed in reality (Reynolds et al. 2009). Given the current uncertainties around how bees respond in different wind conditions, we have not modelled this process, but when clear empirical information becomes available, then the model can be modified accordingly.

Once the final setup of flower patches has been made, the detection probabilities for each of these patches can be determined. This is achieved by simulating the movements of *N_Bees* agents, each representing a single honeybee or bumblebee scout. Starting from the location of the colony, they explore the landscape following an artificial or empirically based flight pattern. The movement is calculated in the procedure *MoveBeesProc*. Each step represents *Gap_*s seconds of real time and the new location of a bee is determined by its turning angle and the step width.

The step width is set in the reporter procedure *DisplacementREP* with *displ_m* 2.96m * Gap_s for honeybees and 3.22m * Gap_s for bumblebees. *Displ_m* is then divided by *Scaling* to transform the real distance into a distance in the Netlogo *world* (*displ_NLpatches*) and multiplied by *DisplacementFactor* (range: 0..10, default: 1) to allow for smaller or larger step widths

We distinguish three flight phases (*flightPhase*) in the bees movement: In *flightPhase* 1, *bees* are heading in an linear vector flight towards a field destination, defined in *destinationList* (e.g. the coordinates of flower patch they visited before) and are shown in green. In *flightPhase* 3 *destinationList* is set to the coordinates of the colony and the *bees* (shown in blue) are heading in an linear vector flight towards the colony. *FlightPhase* 2 describes a small scaled search pattern (although patches can be detected in any *flightPhase*) and bees are shown in white or, if they perform a loop, in orange.

If a bee has no destination (i.e. a white bee during *flightPhase* 2 and not looping), its new flight direction is set in the procedure *TurnNoDestinationProc*. Based on a distribution of turning angles (0 - 180°, bin width 10°) from experimental data, the turning angle to either the right or left side (with equal probabilities) is randomly chosen, e.g. for honeybees (reporter procedure *TurningREP*):

```
if BeeSpecies = "Honeybees"
[
  let randBar random 628 + 1
  if randBar >= 1 and randBar < 92 [set turn 0 + random-float 10]
  if randBar >= 92 and randBar < 144 [set turn 10 + random-float 10]
  ...
  if randBar >= 599 and randBar <= 628 [set turn 170 + random-float 10]
 ]

if random-float 1 > 0.5 [ set turn turn * -1 ]
```

If *RandomWalk* is true, than a random turning angle is chosen from an equal distribution

```
if RandomWalk = true [ set turn random-float 360 ]
```

resulting in an uncorrelated random walk.

The turning angle *turn* is then multiplied with *LinearisationFactor* (range: 0..1, default: 1) to allow for a more linear movement.

If *FixTurningAngle* is true, then the turning angle is set to the fix value *FixRightTurn*, resulting in circular movement of the bees during the searching phase.

A *bee* might then perform a loop: with the probability *TurnToDestinationProb* (default: 2%) its *destinationList* is set to *lastDestinationList*, i.e. it turns toward its previous destination, which can either be a location in the field or - if the bee has never been at a field destination - the location of the colony. They then change their colour to orange until they reach their destination.

If a *bee* has a destination (i.e. it is in *flightPhase* 1 or 3 (green and blue bees) or doing a loop in *flightPhase* 2 (orange bees)), then it turns towards the location of its destination (*destinationList*). If the destination is a location outside the colony and the distance is smaller than the length of a bees' step width, then *lastDestinationList* is updated, *destinationList* is emptied, the bee choses a new, random direction, changes its colour to white, and finally switches to *flightPhase* 2, and (*TurnDestinationProc*):

```
if destinationList !=  []
 [
  if (distancexy item 0 destinationList item 1 destinationList) <
  DisplacementREP and destinationList != list Col_x Col_y
      [
         set lastDestinationList destinationList
         set destinationList []
         set heading random-float 360
         set color white
         set flightPhase 2
      ]
 ]
```

If a *bee* is heading back (*flightPhase* 3) and reaches the *hive* (colony), then *destinationList* is reset and *nowDetectedPatchesList*, a list that contains all patches detected by this bee during its current scouting trip, is emptied.

Furthermore, some data are recorded during the movement of a bee:

- *MaxHiveDisplAllBees_m*: A global variable with the largest distance any of the *bees* ever had to the *hive*.
- *visits*: A Netlogo *patch* (grid cell) specific variable that is increased by 1, whenever a bee is located on this Netlogo patch.
- *lastLocationList*: A *bee* specific variable that records the last xy-position of a bee during *flightPhase* 2. This location is then set to the field destination in the next scouting trip, if the search mode is "last location (individual)".
- *furthestLocationList*, *furthestDistance*: records the xy-coordinates and the distance to the *hive* of the furthest location an individual bee has ever been.

If *RandomTripDuration* is false, then the duration of a trip is defined by *TripDuration_s*, otherwise it is randomly determined with an average duration of *TripDuration_s* (*returningProb* = *Gap_s* / *TripDuration_s*). If a bee ends its scouting trip, it switches into *flightPhase* 3 and heads towards the location of the *hive* (colony) (i.e. *destinationList* is populated with the xy-coordinates of the *hive*):

```
if (RandomTripDuration = false and remainder (ticks * Gap_s) (Gap_s *
                        round  (TripDuration_s / Gap_s)) = 0
    or (RandomTripDuration = true and random-float 1 <= returningProb)
       [
         set destinationList list Col_x Col_y
         set color sky
         set flightPhase 3
       ]
```

The actual movement of a *bee* is implemented as:

```
ask bees with [ nTrips <= MaxTrips ]
  [ fd DisplacementREP
    ...
```

i.e. bees have chosen their direction and now move forward by the distance determined in *DisplacementREP*. Only *bees* who have not performed more than *MaxTrips* scouting trips yet are allowed to move, all others stay in the colony. If the new location of the bee is defined as lake (while the *bee* is white, i.e. in the small scaled search mode) or as border, then the movement is withdrawn and the bee choses randomly a new direction:

```
if (patchColor = LakeColor)
  or (originalColor = BorderColor)
  or (pxcor = max-pxcor) or (pxcor = min-pxcor)
  or (pycor = max-pycor) or (pycor = min-pycor)
   [
     if flightPhase = 2 and destinationList = []
      [
        setxy item 0 lastLocationList item 1 lastLocationList
        set heading random-float 360
] ] ]
```

Whenever a *bee* enters a flower patch (i.e. a grid cell (Netlogo *patch*) with a *patchColor* being defined as food source (potentially: red, green, blue or yellow)) that has not been entered before by this bee in its current scouting round, then the number of this patch is added to the *bees' nowDetectedPatchesList* and *totalDetections* of this flower patch (i.e. of its *PatchStatistic* agent) is increased by 1. If the patch has never been detected before, the patches' *firstDetection_s* is set to the current time.

If the bee has never been at this patch before, the number (*who*) of the patch is added to the *bees' knownPatchesList*. If *ImmediateReturn* is true, then the *bee* switches to *flightPhase* 3 and sets its destination to the colony (irrespective of *TripDuration_s*) (*DetectionProc*):

```
if nTrips <= MaxTrips
  [
     if member? patchColor PatchColoursList
        [
```

```
        let patchNumber flowerPatchID
        if member? patchNumber nowDetectedPatchesList = false
        [
          set nowDetectedPatchesList fput patchNumber
                nowDetectedPatchesList
          ask PatchStatistic patchNumber
             [
               set totalDetections totalDetections + 1
               if firstDetection_s = 0
                   [ set firstDetection_s ticks * Gap_s ]
             ]

   if member? patchNumber knownPatchesList = false
    [
     set knownPatchesList fput patchNumber
     if ImmediateReturn = true
      [
         set destinationList list Col_x Col_y
         set color sky
         set flightPhase 3
      ]
     ]]
```

The exploration of the landscape by the bees finally stops, when *ScoutingPeriod_hrs* is exceeded:

```
   if ticks * Gap_s > ScoutingPeriod_hrs * 3600 [ stop ]
```

<u>Search options:</u>

There are several options (*SearchMode*) how the new field destination is chosen in the reporter procedure *RelocationREP* after a bee has returned to the colony:

"Colony": no field destination is chosen and the bee immediatly switches to *flightPhase* 2:

```
   if SearchMode = "Colony" [ set result []  ]
```

"known flowerpatch (individual)": the bee chooses randomly one location (i.e. Netlogo patch) that is part of a flower patch already detected by this individual bee. Hence, bigger flower patches are proportionally more likely to be chosen:

```
   if SearchMode = "known flowerpatch (individual)"
      [
          let memoKnownPatchesList []
          ifelse empty? knownPatchesList
            [ set result [] ]
            [
              set memoKnownPatchesList knownPatchesList
              ask one-of patches with
                     [ flowerpatchid = one-of memoKnownPatchesList ]
               [ set result list pxcor pycor ]
            ]
        ]
```

"known flowerpatch (recruitment)": similar to "known flowerpatch (individual)", but now, a location in any patch that was detected by any bee can become the new destination. Again, the probability of a flower patch to be chosen is proportional to its size:

```
if SearchMode = "known flowerpatch (recruitment)"
    [
       let memoKnownPatchesList []
       ask patchstatistics
        [
          if totalDetections > 0
           [ set memoKnownPatchesList fput who memoKnownPatchesList ]
        ]
       ifelse empty? memoKnownPatchesList
         [ set result [] ]
         [
            let theChosenPatch one-of memoKnownPatchesList
            ask one-of patches with [flowerpatchid = theChosenPatch
              [ set result list pxcor pycor ] ] ]
```

"furthest location (individual)": the bee chooses the location with furthest distance from the colony it has ever been as its new destination:

```
if SearchMode = "furthest location (individual)"
      [ set result furthestLocationList ]
```

"random location": the bee chooses random location within a distance of *MaxForagingRange*_m from the colony that does not belong to a lake or the borders:

```
if SearchMode = "random location"
     [
        ask one-of patches with
         [ patchColor != LakeColor and
            distancexy Col_X Col_Y * Scaling < MaxForagingRange_m and
               originalColor != BorderColor  ]
         [
            set result list pxcor pycor
         ]
     ]
```

"visited NLpatch (recruitment)": the new destination of a bee is randomly chosen from any location (i.e. Netlogo patch) that was ever visited by any bee and is not placed in a lake. (Note that bees can cross lakes while they are in flightPhase 1 or 3). If no such location exists, then the location of the *hive* (colony) is defined as the new destination:

```
if SearchMode = "visited NLpatch (recruitment)"
[
   ifelse count patches
       with [ visits > 0 and patchColor != LakeColor ] > 0
   [ ask one-of patches
       with [ visits > 0 and patchColor != LakeColor ]
            [ set result list pxcor pycor ] ]
   [ set result list Col_X Col_Y ]
 ]
```

"last location (individual)": the bee returns to the location where it had stopped its previous scouting trip to return to the colony and resumes searching from there:

```
if SearchMode = "last location (individual)"
   [ set result lastLocationList ]
```

If no destination has been chosen, the bee switches to the search mode (*flightPhase* 2), otherwise, flightPhase is set to 1 and the bee heads out to its field destination:

```
ifelse empty? result
    [
      set color white
      set flightPhase 2
    ]
    [
      set color green
      set flightPhase 1
    ]
```

If SearchMode is set to "mixed strategy (individual)" or "mixed strategy (recruitment)", then the search mode is randomly chosen in the procedure *MoveBeesProc* from a list with whenever a new field destination of a bee is determined:

```
ask bees
   [ ...
     if SearchMode = "mixed strategy (individual)"
       [
         set MixedStratInd true
         set SearchMode one-of
          [ "colony" "known flowerpatch (individual)" "furthest
             location (individual)" "last location (individual)" ]
          ]

     if SearchMode = "mixed strategy (recruitment)"
       [
         set MixedStratCol true
         set SearchMode one-of
          [ "colony" "visited NLpatch (recruitment)" "known
             flowerpatch (recruitment)" "furthest location
             (individual)" "last location (individual)" ]
          ]
```

The variables *MixedStratInd* and *MixedStratCol* are required to finally set SearchMode back to its initial "mixed strategy" choice:

```
if MixedStratInd = true
   [ set SearchMode "mixed strategy (individual)" ]
if MixedStratCol = true
   [ set SearchMode "mixed strategy (recruitment)" ]
```

II. A module for creating and modifying a landscape of flowering patches.

If no input file for a forage map is provided, then the color (*orignalColor*) of all grid cells (Netlogo *patches*) is set to grey. The user can then define flower patches with the help of the "Artificial landscape creator". Flower patches can either be drawn with the mouse directly on the map, after a colour and a brush size was chosen from *SetColour* and *BrushSize*. Alternatively, the user can define (circular) flower patches by their size (*SetRadius_m*), their distance (*SetDistanceToCentre_m*), and their direction (*SetDirection_deg*) from the colony. Both methods of creating patches can be combined and also be used, if a forage map was uploaded. After all patches are created or modified, the setup process has to be repeated by

pressing the "Update" button. Note that the borders are identical with the borders of the Netlogo world, if no crop map was imported.

III. Define food flow and create inputfile for BEEHAVE colony model

The Landscape module can also be used to create an input file (*NameOutfile*) for the BEEHAVE colony model. By pressing the "Analyse & Outfile" button, the crop map is imported and analysed as described above and the bees explore the landscape as defined by in the interfaces' "Search mode" options. At the end of the run, the results are written in a file (*NameOutfile*). This file contains information on size, location, nectar and pollen availability and detection probabilities of each identified flower patch. As nectar and pollen availability can change with the season, all information are provided for each day of the year, hence, the file consists of N flower patches x 365 + 1 (header) lines. The 15 columns of the file are:

1. day: the day of year (1..365)
2. id: the id of a flower patch (0.. N-1 flower patches within *MaxForagingRange_m*)
3. oldPatchID: the original id (*who*) of a flower patch (i.e. the number shown in the map of the interface) (identical with id, unless flower patches are removed due to a limiting *MaxForagingRange_m*)
4. patchType: information for the user about the crop or flowers in the patch (e.g. "OSR" or "RedPatch")
5. distance_m: closest distance [m] of the patch to the hive (colony)
6. xcor: distance [m] on x-axis to the hive (colony)
7. ycor: distance [m] on y-axis to the hive (colony)
8. size_sqm: size of the flower patch [m^2]
9. quantityPollen_g: amount of pollen [g] offered at the patch today
10. concentration: sugar (sucrose) concentration [mol/l] of the nectar
11. quantityNectar_l: amount of nectar [l] offered at the patch today
12. calculatedDetectionProb_per_trip: the probability that a scout finds the flower patch during a single scouting trip, calculated on the basis of size and distance of the patch
13. modelledDetectionProb_per_trip: the probability that a scout finds the flower patch during a single scouting trip as an output of the simulation run
14. nectarGathering_s: handling time [s] for nectar, i.e. the time a bee has to spent in a patch to fill its crop
15. pollenGathering_s: handling time [s] for pollen, i.e. the time a bee has to spent in a patch collect a pollen load

Data on patch type, quantity of nectar [ml/ m^2] and pollen [g/ m^2], handling times [s], nectar concentration [mol/l] have to be provided by the user (interface: "Definition of flower

patches"). Flower patches only offer nectar and pollen during a certain period of the year, defined by *Start_(patchtype)* and *Stop_(patchtype)*.

IV. Display of foraging activities, modelled in BEEHAVE

The output file *NameOutfile* can now be used as an input file for the BEEHAVE colony model. BEEHAVE also offers the options, to write the nectar and pollen visits of all patches for each day of one year into a file (default name: "Input_1-2_Foraging.txt"). This file can then be uploaded by the Landscape module (interface: "Show foraging data") to display either the nectar and/or pollen visits on the map for each day of a year. Various buttons allow the user to move forwards or backwards through the dataset, to show a specific date or to run a slide show.

REFERENCES

Becher, M. A., Grimm, V., Thorbek, P., Horn, J., Kennedy, P. J., & Osborne, J. L. (2014). BEEHAVE: a systems model of honeybee colony dynamics and foraging to explore multifactorial causes of colony failure. *Journal of Applied Ecology*, 51(2), 470-482.

Biesmeijer, J. C., & Seeley, T. D. (2005). The use of waggle dance information by honey bees throughout their foraging careers. *Behavioral Ecology and Sociobiology*, 59(1), 133-142.

von Frisch, K. (1967). The dance language and orientation of bees. Cambridge, MA: Harvard University Press.

Giurfa, M., Vorobyev, M., Kevan, P., & Menzel, R. (1996) Detection of coloured stimuli by honeybees: minimum visual angles and receptor specific contrasts. *Journal of Comparative Physiology A*, 178, 699-709.

Grüter, C., & Farina, W. M. (2009). The honeybee waggle dance: can we follow the steps?. *Trends in Ecology & Evolution*, 24(5), 242-247.

Grimm, V., Berger, U., Bastiansen, F., Eliassen, S., Ginot, V., Giske, J., Goss-Custard, J., Grand, T., Heinz, S.K., Huse, G., Huth, A., Jepsen, J.U., Jørgensen, C., Mooij, W.M., Müller, B., Pe'er, G., Piou, C., Railsback, S.F., Robbins, A.M., Robbins, M.M., Rossmanith, E., Rüger, N., Strand, E., Souissi, S., Stillman, R.A., Vabø, R., Visser, U. & DeAngelis DL (2006) A standard protocol for describing individual-based and agent-based models. *Ecological Modelling*, 198, 115-126.

Grimm, V., Berger, U., DeAngelis, D.L., Polhill, G., Giske, J. & Railsback, S. F. 2010 The ODD protocol: a review and first update. *Ecological Modelling*, 221, 2760-2768.

Heran, H., & Lindauer, M. (1963). Windkompensation und Seitenwindkorrektur der Bienen beim Flug über Wasser. *Zeitschrift für vergleichende Physiologie*, 47(1), 39-55.

Pahl, M., Zhu, H., Tautz, J., & Zhang, S. (2011). Large scale homing in honeybees. PLoS One, 6(5), e19669.Pyke, G. H. (1984). Optimal foraging theory: a critical review. *Annual review of ecology and systematics*, 523-575.

Reinhard, J., & Srinivasan, M. V. (2009) The role of scents in honey bee foraging and recruitment. In: Jarau, S., & Hrncir, M. (eds) Food exploitation by social insects. Ecological, behavioral, and theoretical approaches. CRC Press, Boca Raton, pp 165–182.

Riley, J. R., Reynolds, D. R., Smith, A. D., Edwards, A. S., Osborne, J. L., Williams, I. H., & McCartney, H. A. (1999) Compensation for wind drift by bumble-bees. Nature, 400, 126-126.

Riley, J. R., Greggers, U., Smith, A. D., Reynolds, D. R., & Menzel, R. (2005) The flight paths of honeybees recruited by the waggle dance. Nature, 435, 205-207.

Seeley, T. D. (1983). Division of labor between scouts and recruits in honeybee foraging. Behavioral ecology and sociobiology, 12(3), 253-259.

Towne, W. F., & Gould, J. L. (1988). The spatial precision of the honey bees' dance communication. *Journal of Insect Behavior*, 1(2), 129-155.

Weidenmüller, A., & Seeley, T. D. (1999). Imprecision in waggle dances of the honeybee (Apis mellifera) for nearby food sources: error or adaptation? *Behavioral Ecology and Sociobiology*, 46(3), 190-199.

Wilensky, U. (1999). NetLogo. *http://ccl.northwestern.edu/netlogo/.* Center for Connected Learning and Computer-Based Modeling, Northwestern University, Evanston, IL.

Wright, G. A., & Schiestl, F. P. (2009) The evolution of floral scent: the influence of olfactory learning by insect pollinators on the honest signalling of floral rewards. Functional Ecology, 23, 841-851.
