# Supplementary material for: BEESCOUT: A model of bee scouting behaviour and a software tool for characterizing nectar/pollen landscapes for BEEHAVE
Source: Ecol Modell. 2016 Nov 24;340:126–33. doi: 10.1016/j.ecolmodel.2016.09.013 (PMC5070411; doi:10.1016/j.ecolmodel.2016.09.013)
Supplement: Supplementary file 3 — S3: A user's manual for the BEESCOUT software tool. [file mmc3.pdf]

BEESCOUT

User Manual

Contents

1. BEESCOUT: an introduction 2

1.1. Overview of Interface 2

2. Setup map 4

2.1. Import crop map 4

If this is the first time you have used BEESCOUT, you can now run the model using the default settings to make yourself familiar with the output. To do this, go straight to section 3 of this manual. Once you are familiar with the model and wish to use your own scenarios, details of how to modify the landscape, set up forage patch attributes, and how to define bee colony type, size and behaviour are in sections 2.1.1 – 2.4 5

2.1.1. Set scaling 5

2.2. Create or modify a landscape 6

2.3. Define food flow of the patches 7

2.4. Search options and bee movement 8

3. Running the Model and Output options 9

3.1. Map Display 10

3.2. Output plots and fields 10

3.3. Output a File for BEEHAVE 11

4. Display simulated visitation rates from BEEHAVE 11

5. Summary tables 13

5.1. Input options 13

5.1.1. Setup map 13

5.1.3. Advanced initialisations 14

5.1.4. “Input field” 14

5.1.5. “Switch” 16

5.1.6. “Chooser” 17

5.1.7. “Slider” 19

5.2. Output options 20

5.2.1. Map display 20

5.2.2. Output plots and fields 21

5.2.3. Output file for BEEHAVE 21

7. List of tables 22

1. BEESCOUT: an introduction

BEESCOUT is a spatially explicit, individual-based model, used to determine detection probabilities of food sources for scouting bees. The model is able to identify the size and location of food patches and obstacles according to their colour, in a landscape of choice, by analysing a two-dimensional map, either read in as an image file or created within the program. The software can also produce input files for the honey bee model BEEHAVE (Becher et al. 2014) to allow the simulation of foraging in a specified landscape. In return BEESCOUT can display the simulated foraging activities imported from BEEHAVE on its map.

During a simulation bees explore the landscape, either in a linear flight pattern (when heading to a destination or returning to the colony) or in a smaller scaled flight pattern (when searching the landscape, or during their very first scouting trip). The movement of bees in the search phase is determined by a turning angle (i.e. the change in direction) and step length. Default values for these are based on empirical data from radar tracked bees, whereby turning angle is drawn from a random distribution.

A simulation will stop when the total time allowed for scouting is exceeded. The detection probabilities for the flower patches are then calculated and the results saved in an output file.

In this manual we explain what you see on the model interface when you open BEESCOUT, and then how to set up a landscape with a colony of bees. We explain the different options available for specifying the type and behaviour of the bees, and then show how the model runs, and what the outputs display. Options for creating input files for BEEHAVE, and displaying its output, are described. The last section of the manual contains tables of all the standard and advanced options for the set up, input and outputs.

We recommend that when you first run the BEESCOUT model, you simply choose the initial default settings (i.e. make sure the model and all input files are unzipped and in the same folder, press the buttons "Default (Honeybees)" (under section"Setup Map") and then the button "BB" (Interface section 2 ("My scenarios") for a small colony of 30 bumblebees placed in a landscape called Map_Osborne.jpg with blue patches denoting where flowers with nectar and pollen are available) so that you become familiar with the interface and outputs.

1.1. Overview of Interface

After you have started BEESCOUT, you will discover an interface tab comprising several initialisation and output options (use Control/– or control/+ to zoom the view). These are used to determine the number, location, size and detection probability of food sources for a bee colony, which can be input into the BEEHAVE colony model.

These options are explained in the following sections of this user manual:

- ◊ Setup map
- ◊ Search options and bee movement

- ◊ Running the model and Output options
- ◊ Display visitation rates from BEEHAVE

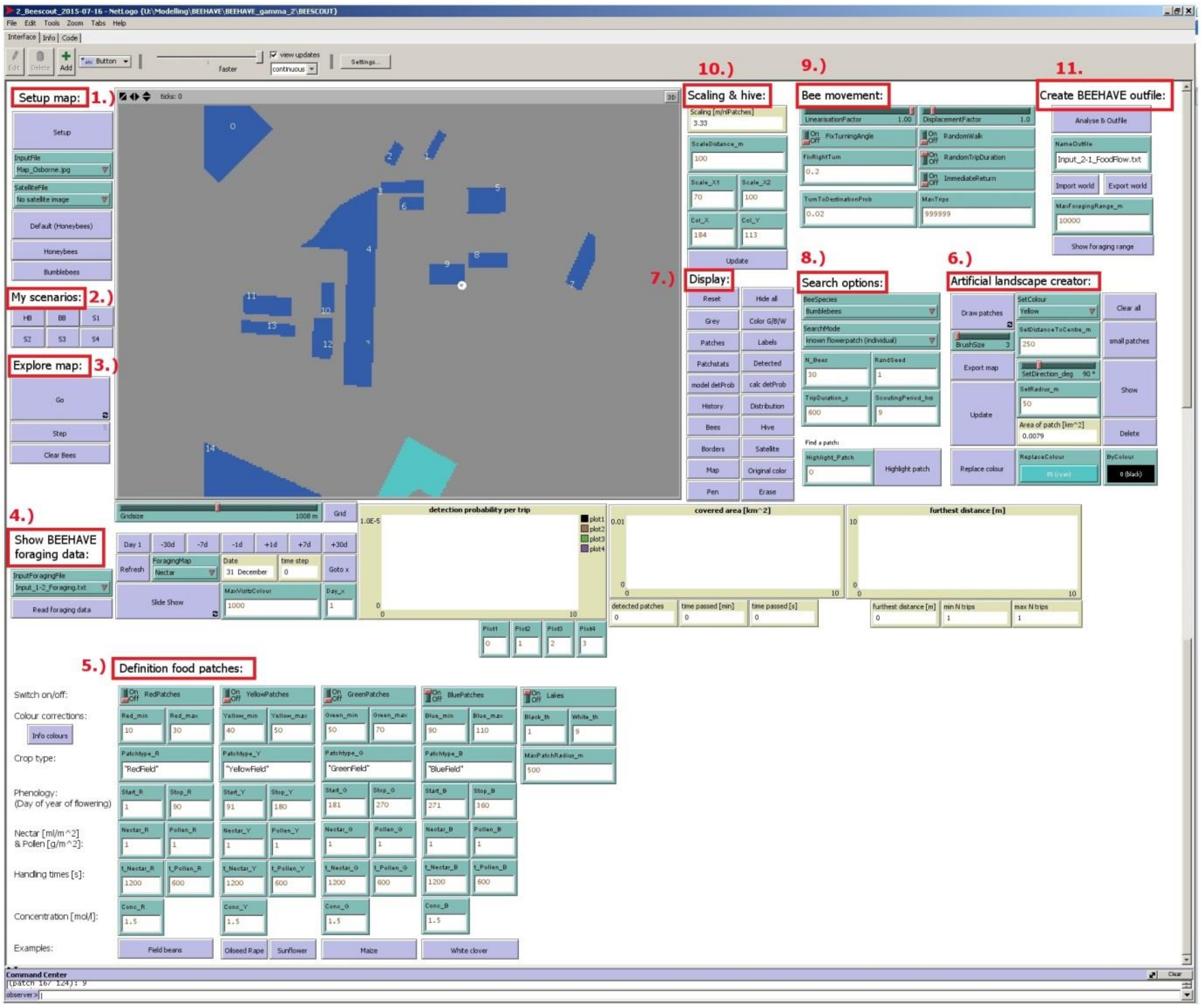

Table 1. Different sections of the BEESCOUT user interface (also highlighted in red above) which will be referred to in the following sections

	Interface section	Description
1	**Setup map**	Import crop map, pre-defined scenarios
2	**My scenarios**	Set up (pre)defined scenarios
3	**Explore the map**	Begin model simulation
4	**Show BEEHAVE foraging data**	Display foraging patterns simulated in BEEHAVE
5	**Definition food patches**	Define flower patches (nectar, pollen, handling times etc.)
6	**Artificial landscape creator**	Create your own landscape
7	**Display**	Adjust map display
8	**Search options**	Change between honeybees and bumblebees and the mode by which they explore forflower patches
9	**Bee movement**	Change characteristics of bee flight when she searches the landscape
10	**Scaling & hive position**	Location of hive and scaling of map
11	**Create BEEHAVE Outfile**	Creating an output file for BEEHAVE

The Netlogo toolbar allows you to control the speed of simulation runs and the viewing of updates. Use the speed slider to control the speed of the model run, and use the chooser to adjust the frequency of viewing updates (from continuous to tick based; a "tick" refers to a time step in the model, which usually represents 3 seconds). Thus, "continuous updates" means that the Netlogo view will be updated many times a second, whereas "on-tick updates"means results in Netlogo view are only updated when the tick counter proceeds.

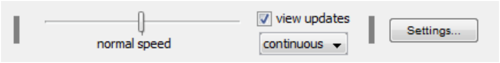

2. Setup map

During the initialisation process a crop map is either imported as an image file or created within the model. If a crop map is provided it can be updated using the artificial landscape creator.

2.1. Import crop map

1. Open a crop map in BEESCOUT. You can choose one already saved in the models folder or one of your own maps, as long as it is saved in an image file format (BMP, JPG, GIF, or PNG). To add your own map, right click on the input file drop down list, click edit and add the filename (+extension) in quotes e.g. "Myownmap.jpg". Additionally, you can import a satellite image, to better relate the crop map to the real landscape.

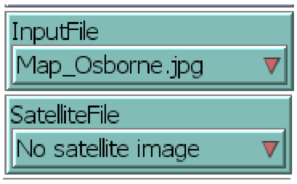

Interface section 1: select crop map and satellite image (if available)

2. Press "Setup" (**wait whilst button is black**; it can take a while, up to a few minutes).

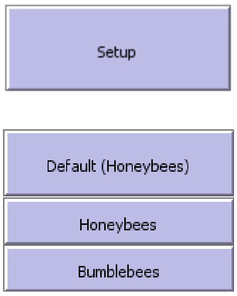

Stage 2 setup map

If this is the first time you have used BEESCOUT, you can now run the model using the default settings (just press the button "BB" under "My Scenarios") to make yourself familiar with the output. To do this, go straight to section 3 of this manual. The button "Default (Honeybees)" sets all input variables to their default values (except of RandSeed and Plot1-4), which represents a honeybee colony in an empty landscape. The button "Honeybees" sets only bee related input variables to the honeybee specific values and "Bumblebee" to the bumblebee specific values. The buttons "HB" and "BB" (Interface section 2, "My Scenarios") set up a honeybee or bumblebee colony in a real landscape with a defined colony location under otherwise default settings. Once you are familiar with the model and wish to use your own scenarios, details of how to modify the landscape, set up forage patch attributes, and how to define bee colony type, size and behaviour are in sections 2.1.1 – 2.4.

2.1.1. Set scaling

Once a map is imported you will need to set the scale and location of the colony (Interface elements "Scaling & hive position").

3. To set the scale you will need the x coordinates of two known locations on the map. To find these, right click once on the map in each location (the x coordinate will be the first number after "inspect patch"). Enter these in "Scale_X1" and "Scale_X2" and the real distance between the two (the "Grid" button could help here) in the "ScaleDistance_m" input fields.

4. To set the location of the colony you will need an x and y coordinate of its location, to do this right click where the colony is on the map once (the x coordinate is the first and the y coordinate the second number after "inspect patch"). Enter these values into "Col_X" and "Col_Y".

5. If you wish, you can set up a new button in the "My Scenarios" section (right click on an area of white and click “Button”), **or** modify a button of "My Scenarios" (right click on e.g. “S1” and click “edit") to automatically scale your map or set up your own scenarios for future use.

   set InputFile "Myownmap.jpg"
   set ScaleDistance_m xxx
   set Scale_X1 xxx
   set Scale_X2 xxx
   set COL_X xxx
   set COL_Y xxx
   (replace xxx with correct values)

6. Press “Update scaling & hive”. Be patient! Depending on the number of patches and ScaleDistance_m this might take some time.

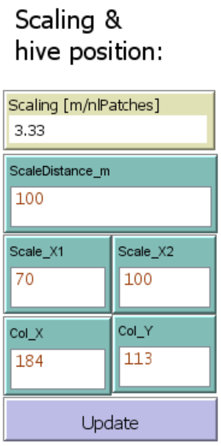

Interface Section 10: Set the scale and location of the colony, update map.

2.2. Create or modify a landscape

7. If the map you have imported needs modifying, or you want to creat our own, then you can do so with the **artificial landscape creator** (Interface section 6).

8. To draw food patches directly onto the map using the mouse, click “Draw patches”. As long as this button is black, you are in “patch drawing mode”. To end this mode, click this button again. Specify your choice of colour from the drop down list “SetColour”, draw on the map to define the location of one or more new patches (make sure the speed slider is set to "normal" and "N_Bees" is small) Finally, click "Update map" to start the setup again and create the patch(es)

9. To generate a patch of a certain size, direction and distance from the colony, specify your choice of colour from the drop down list. Set the size of the patch “SetRadius_m”, its

distance “SetDistanceToCentre_m” and direction “SetDirection_deg” from the colony. Press “Show” to add or "Delete" to remove this patch again.

10. To generate randomly distributed forage patches of minimal size specify your choice of colour from the drop down list ("SetColour") and press “Add sources”. Similarly, "Add by colour" distributes additional small food sources, but only on those patches defined by "ReplaceColour". E.g. if "ReplaceColour" is set to 45 (yellow) and "SetColour" is set to red, then pressing "colour patches" would add small red patches on yellow areas. This might be useful to add small food sources to certain habitat types.

11. If necessary, you can change the colours once they have been drawn using the “Replace colour” buttons.

12. Press “**Update map**” when you are finished, and if you wish “Export map” to create an image file of the map. *Remember:* updating the map can take some time (the area of the map already analysed is presented in green to show the progress).

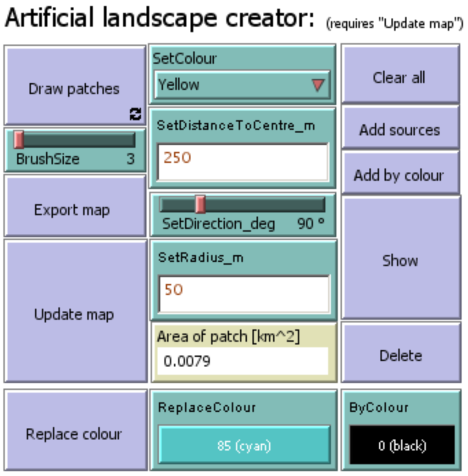

Interface Section 6: Create your own landscape or improve an existing one, update map.

2.3. Define food flow of the patches

13. Define food flow in the landscape, by turning different colour patches on and off. This determines whether they can be found while the bees are searching. The default+BB setting only has blue patches switched on, so these are the only ones that bees will detect.

14. You can also set the quantity of nectar and pollen and concentration of nectar available at the patches, according to their colour. Handling times of nectar and pollen and phenology of each patch can also be defined (Table 4). Note, however, that the nectar and pollen flow in the patches **does not** affect the searching behaviour of the bees in the BEESCOUT model, but is required to generate an input file for the honeybee model BEEHAVE.

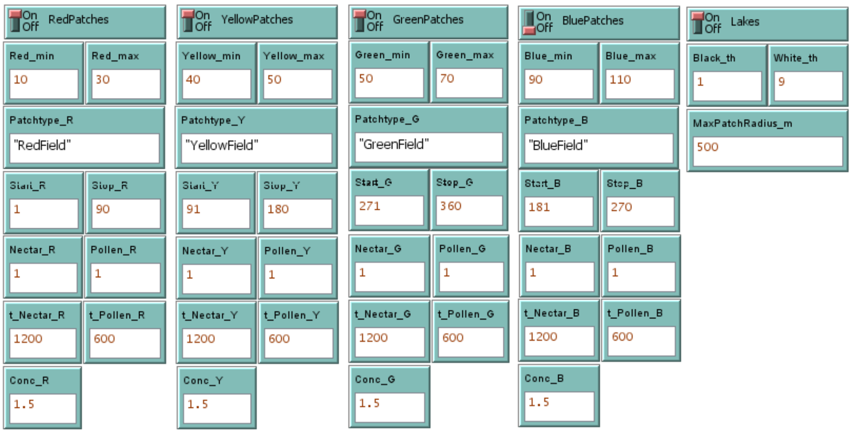

Interface Section 3: define food flow in the landscape

15. Create barriers which bees are unable to cross (during their searching phase), by turning "Lakes" on. If switched on, black areas are interpreted as lakes during the Setup process. Remember, if you alter any of the settings to define patches, you need to press the **setup** button again before running the model.

16. To make sure that the areas of food patches identified by BEESCOUT match closely the actual patch areas, you can fine tune the colour shades defining red, blue, yellow and green by setting suitable values for the "Min" and "Max" colour range under "colour corrections". Black/White_th defines the thresholds for black and white. "Info colour" shows the NetLogo colour scheme to help you making your choice.

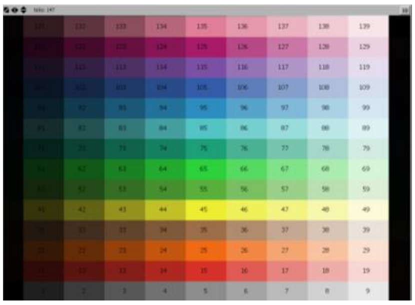

Interface Section 5: Adjust colour of patches so that they are grouped into red, yellow, green, blue and black.

2.4. Search options and bee movement

17. Choose to simulate honeybees or bumblebees (choose honeybees for incorporation into BEEHAVE). Choose a search mode (p. 18), from the drop down options.

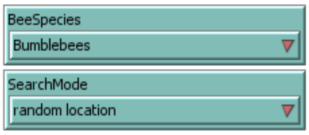

Interface Section 8: Choose bee species and search mode

18. Input the number of bees ("N_Bees") in the colony, duration of a foraging trip “TripDuration_s”, the duration of the simulation “ScoutingPeriod_hrs”, and the random seed ("RandSeed")(Table 4). If RandSeed is set to values other than 0, it acts as input for the pseudo-random number generator so that runs can be reproduced.

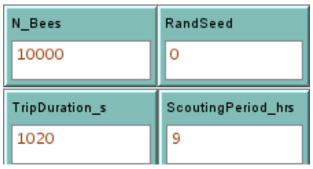

Interface Section 9: Input the number of bees, trip duration, simulation duration and the random seed

19. Specify how the bees explore the landscape. Default values are based on empirical data from radar tracked honeybees. Alternatively, they can be changed to other flight patterns that have been discussed in relation to exploring animals for example:
    - To create spiral movement of bees turn “FixTurningAngle” on and specify the angle of the turn using “FixRightTurn”. Adjust the linearization factor to make movement more linear. Alternatively, turn “RandomWalk” on to ensure uncorrelated, random movement.
    - Adjust the probability that bees will loop around their field destination, using the “TurnToDestinationProb”.
    - Adjust the step length of the bees flight by adjusting the “DisplacementFactor”
    - Turn random trip duration off and set trip time to a specified value. If “RandomTripDuration” is on, then the duration of each individual trip is randomly determined, with an average duration of TripDuration_s seconds.
    - Select “ImmediateReturn” to have bees return to the hive as soon as they have detected a patch they had never visited before (or latest at the end of the trip).

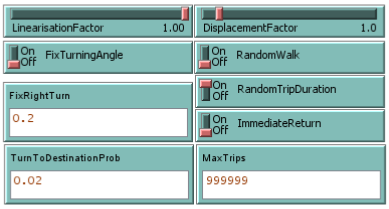

Interface Section 4: Choose settings for bee movement

Once setup, the crop map is analysed to determine the number, size and type of flower patches (red, green, blue, or yellow), as well as their distance to the hive. Finally, virtual bees are created when you press **Go**.

3. Running the Model and Output options

The BEESCOUT interface displays bees foraging in the landscape on a central map. As well as, several output plots and monitors to closer examine the detectability of patches and foraging behaviour of the bees in real time.

3.1. Map Display

20. To start (and pause) the model press "**Go**". Alternatively press "**Step**" to see the model progress for each time step (3 seconds). Use "**Clear Bees**" to reset the number of patch detections to zero and re-create bees. As soon as a simulation is started the map will show bees of different colours (Table 8) exploring the landscape (progress of the simulation can be seen on the monitor "Progress of simulation run [%]")).

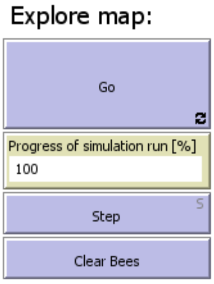

Interface Section 3: Start the simulation

21. Play with the display options (Table 8). For example, use the "Pen" to show tracks and "Erase" to remove.

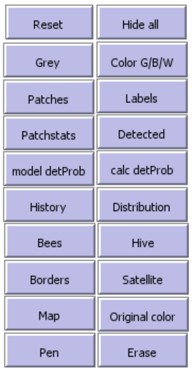

Interface Section 7: Change display options

3.2. Output plots and fields

While the model is running:

22. Take a look at output plots (Table 9). In the first graph, plots 1-4 define the numbered patches that are shown on the graph. Use "Highlight patch" to find a numbered patch on the map and show it on the graph.

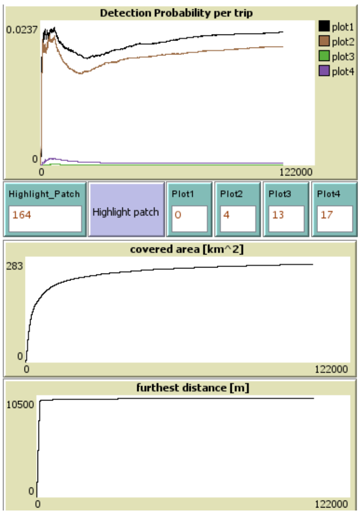

Observe output plots

23. Other output fields (Table 10) (currently below the search options) show the minimum and maximum number of trips the bees have performed so far.

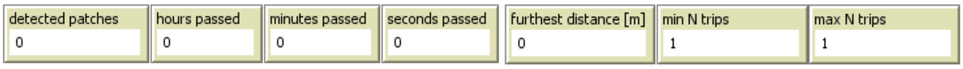

Observe output fields

3.3. Output a File for BEEHAVE

1. Specify a file name and press the “Analyse & Outfile” button. The crop map ("InputFile") is automatically imported, analysed and represented on the central foraging map as before. The bees then start to explore the landscape and at the end of the run, the results are written in a file which contains information on size, location, nectar and pollen availability and detection probabilities of each identified food patch (Table 11). This file serves as input file for the BEEHAVE model.

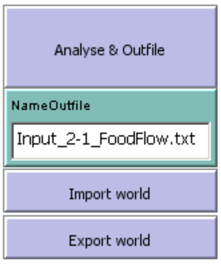

Interface Section 11: Create an output file for BEEHAVE

4. Display simulated visitation rates from BEEHAVE

Finally, BEESCOUT also has the option to visualise the foraging patterns of bees generated from a BEEHAVE simulation. BEEHAVE will record all nectar and pollen visits at all patches and will write these data to a file. This requires an input file (see section 3.3) to be created for and run in BEEHAVE

and in return an “InputForagingFile” to be created by BEEHAVE for BEESCOUT. Make sure you use the same map to display simulated visitation rates from BEEHAVE as when creating the input file for BEEHAVE in the first place.

2. Select the file and choose whether you would like to display nectar, pollen or all visits. Press “Read foraging data” and either “Slide show” or +/- x days to show the visitation rates at all patches for nectar and/or pollen foragers for each day of a 365d simulation.

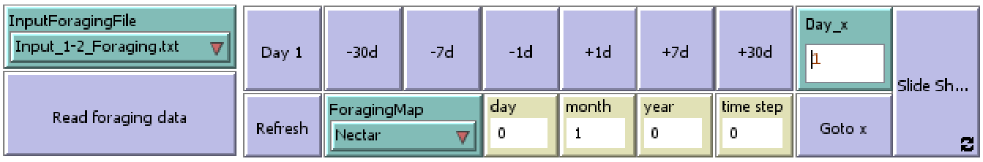

Interface Section 4: Visualise foraging patterns of honeybees generated from a BEEHAVE simulation

Users of the new Beehave_BeeMapp(2015) version can create the foraging data file for BEESCOUT by pressing (in Beehave) the button "1-2 foraging file" (in the section "FILES - out"). Users of the original Beehave(2013) version can amend the model by editing the button "1-3 foraging file" (in the section "Special output") or create a new button (right mouse click on white area of the Beehave interface, "Button"). Copy the following code to the button's "Commands" box and choose a "Display name":

```
set ReadInfile TRUE
set StopDead false
Setup
let filename "Input_1-2_Foraging.txt"
if file-exists? filename [ file-delete filename ]
file-open filename
file-print count flowerPatches
file-print "day who nectarVisits pollenVisits"
repeat 365 [
  startProc
  foreach sort flowerpatches [ ask ? [
    file-type day file-type " "
    file-type oldPatchId file-type " "
    file-type nectarVisitsToday file-type " "
    file-type pollenVisitsToday file-print " "
   ] ]
] ; end repeat
file-close
user-message "Input file ('Input_1-2_Foraging.txt') was created for the external landscape
module BEESCOUT"
```

5. Summary tables

5.1. Input options

The BEESCOUT interface offers a large number of input options using “Input fields”, “Switch” and “Chooser” buttons. These define the parameter settings, input data and a number of adjustment options for foraging processes. Thus, the following tables in this section give a review of the various input options in order to clarify these complex model initialisation settings. Moreover, initial settings (default values) of all input variables are demonstrated.

5.1.1. Setup map

The interface tab of BEESCOUT provides several setup and running buttons that initiate and start the simulation run as well as offering the option to choose pre-defined scenarios.

The **Setup** button initiates the model settings called by the parameterisation procedure. It is only required if a new map is loaded, modified or the scaling or position of hive is changed, i.e. anything that changes the landscape in relation to the colony. The model can be run stepwise or continuously.

Furthermore, you can start pre-defined scenarios (Table 2).

Table 2 pre-defined scenarios available in BEESCOUT

Pre-defined scenario	Function	Interface section
Default (Honeybees)	All input variables are reset to their default values, apart from RandSeed and Plot1-4; no InputFile (map) loaded	Setup map (1)
Honeybees	Sets (only) the bee related variables to their "honeybee" value	Setup map (1)
Bumblebees	Sets (only) the bee related variables to their "bumblebee" value	Setup map (1)
HB	Sets default conditions (honeybees) with a crop map (RealLandscape.jpg) loaded	My scenarios (2)
BB	Sets a bumble bee colony with a crop map (Map_Osborne.jpg) loaded under otherwise default conditions	My scenarios (2)
Field beans	Sets phenology and nectar & pollen production to the "Field beans" values	Definition food patches (5)
Oilseed rape	Sets phenology and nectar & pollen production to the "Oilseed rape" values	Definition food patches (5)
Sunflower	Sets phenology and nectar & pollen production to the "Sunflower" values	Definition food patches (5)
Maize	Sets phenology and nectar & pollen production to the	Definition food patches (5)

	"Maize" values	
White clover	Sets phenology and nectar & pollen production to the "White clover" values	Definition food patches (5)

5.1.3. Advanced initialisations

You can choose several advanced model initialisations and outputs by clicking defined buttons during a model run (Table 3).

Table 3 create outfiles options

Button	Function	Interface section
Analyse & Outfile	Map is imported and analysed and bees explore the landscape as defined by the interfaces" "Search mode" and "bee movement" options. At the end of a run, the results are written in a file for the BEEHAVE model	Create BEEHAVE outfile (11)
Import world	Imports an exported NetLogo "World" file	Create BEEHAVE outfile (11)
Export world	Writes the current values of all variables, plots etc into a NetLogo "World" file	Create BEEHAVE outfile (11)
Show foraging range	Highlights the foraging range of bees	Show BEEHAVE foraging data (4)

5.1.4. "Input field"

Input fields allow you to initialise and change the most important parameter settings of foraging processes (Table 4).

Table 4 input fields

Variable	Definition	Initial Setting	Interface
RandSeed	Initial value of the pseudo-random number generator; undefined if set to 0.	not set	Search options (8)
N_Bees	Initial colony size	10000	Search options (8)
TripDuration_s	Duration of a scouting trip	1020	Search options (8)
ScoutingPeriod_hrs	Total duration of the simulation	9	Search options (8)
FixRightTurn	Set turning angle for every step	0.2	Bee movement (9)
TurnToDestinationProb	Probability that a bee will loop before a patch	0.02	Bee movement (9)
MaxTrips	Maximum number of scouting trips	999999	Bee movement (9)

SetDistanceToCentre_m	Distance of patch from the colony	3500	Artificial landscape creator (6)
SetRadius_m	Radius of patch	500	Artificial landscape creator (6)
Highlight_Patch	Patch number to be highlighted	0	Artificial landscape creator (6)
Plot1<br>Plot2<br>Plot3<br>Plot4	Define the number patch for which the detection probability is plotted	not set	Artificial landscape creator (6)
Day_x	Defines the number of days simulated by the "Goto x" button	1	Show BEEHAVE foraging data (4)
MaxForagingRange_m	Maximum foraging distance of the colony	10000	Show BEEHAVE foraging data (4)
MaxVisitsColour	allows to adjust the display of simulated foraging visits to the foraging activity	1000	Show BEEHAVE foraging data (4)
red_min<br>yellow_min<br>green_min<br>blue_min	Minimum Netlogo colour value for a grid cell to be interpreted as red, blue etc.	10<br>40<br>50<br>90	Definition food patches (5)
red_max<br>yellow_max<br>green_max<br>blue_max	Maximum Netlogo colour value for a grid cell to be interpreted as red, blue etc.	30<br>50<br>70<br>110	Definition food patches (5)
Patchtype_R<br>Patchtype_Y<br>Patchtype_G<br>Patchtype_B	Name given to patch/ crop type	"RedField"<br>"YellowField"<br>"GreenField"<br>"BlueField"	Definition food patches (5)
Start_R<br>Start_Y<br>Start_G<br>Start_B	First day of flowering in the year	1<br>91<br>271<br>181	Definition food patches (5)
Stop_R<br>Stop_Y<br>Stop_G<br>Stop_B	Last day of flowering in the year	90<br>180<br>360<br>270	Definition food patches (5)
Nectar_R<br>Nectar_Y<br>Nectar_G<br>Nectar_B	Quantity of nectar available at each patch [ml/ m^2]	1<br>1<br>1<br>1	Definition food patches (5)
Pollen_R<br>Pollen_Y<br>Pollen_G<br>Pollen_B	Quantity of pollen available at each patch [g/ m^2]	1<br>1<br>1<br>1	Definition food patches (5)
T_Nectar_R<br>T_Nectar_Y<br>T_Nectar_G	Time spent handling nectar [s] at each patch of that type	1200<br>1200<br>1200	Definition food patches (5)

T_Nectar_B		1200	
T_Pollen_R<br>T_Pollen_Y<br>T_Pollen_G<br>T_Pollen_B	Time spend handling pollen [s] at each patch of that type	600<br>600<br>600<br>600	Definition food patches (5)
Conc_R<br>Conc_Y<br>Conc_G<br>Conc_B	Sucrose concentration of nectar at each patch of that type [mol/l]	1.5<br>1.5<br>1.5<br>1.5	Definition food patches (5)
Black_th	Max. threshold of dark colours to be interpreted as black	1	Definition food patches (5)
White_th	Min. threshold of light colours to be interpreted as white	9	Definition food patches (5)
MaxPatchRadius_m	Defines the maximum radius a patch can have, i.e. any patch of the same colour which is larger than this value will be divided into two or more sub patches	500	Definition food patches (5)
ScaleDistance_m	Real distance between two X coordinates on the map	3000	Scaling and hive position (10)
Scale_X1	X coordinate for reference point 1	181	Scaling and hive position (10)
Scale_X2	X coordinate for reference point 2	210	Scaling and hive position (10)
Col_X	X coordinate of colony	160	Scaling and hive position
Col_Y	Y coordinate of colony	106	Scaling and hive position (10)
NameOutfile	Specify a name for the out file to be used as an input file for the BEEHAVE colony model.	"Input_2-1_FoodFlow.txt"	Create BEEHAVE out file (11)

5.1.5. "Switch"

The BEESCOUT interface tab includes a large number of switches for Boolean variables. Here important input data and settings are specified (Table 5).

Table 5 switch variables

"Switch" variable	Definition	Default	Interface
FixTurningAngle	Sets turning angle (on) resulting in circular movement	Off	Bee movement (9)
RandomWalk	Random turning angle is chosen from equal distribution resulting in	Off	Bee movement (9)

	uncorrelated random walk.		
RandomTripDuration	Trip duration is randomly determined (on), or set to a specified value (off)	On	Bee movement (9)
ImmediateReturn	Bee switches to flight phase 3 (Table 8), and returns to hive when it detects a new patch	Off	Bee movement (9)
RedPatches	Turns red patches on or off (change requires new setup)	Off	Definition food patches (5)
YellowPatches	Turns yellow patches on or off (change requires new setup)	Off	Definition food patches (5)
GreenPatches	Turns green patches on or off (change requires new setup)	Off	Definition food patches (5)
BluePatches	Turns blue patches on or off (change requires new setup)	Off	Definition food patches (5)
Lakes	Turns lakes on or off (change requires new setup)	Off	Definition food patches (5)

5.1.6. "Chooser"

Additionally, the BEESCOUT interface tab offers several chooser input options. These allow for different example landscapes to be input into the model, as well as different search modes of the bees to be specified (Table 6).

Table 6 chooser variables

"Chooser" variable	Definition	Options	Interface
InputFile	Choose a crop map to be imported. The user has to set reference points and the real distance for the correct scaling. If no input file for a crop map is provided, then the colour (orignalColor) of all Netlogo patches is set to grey. The user can then define food patches with the help of the "Artificial landscape creator".	"No input file"<br>"RealLandscape.jpg"<br>"Map_Osborne.jpg"<br>"MapImageUpdate.png"<br>"MapImage.png"	Setup map (1)
SatelliteFile	Import satellite image to better relate the crop map to the real landscape	No satellite image	Setup map (1)
InputForagingFile	Choose an input file created by a	Input_1-2_Foraging.txt	Show BEEHAVE

	simulation run of BEEHAVE to visualize the foraging patterns the bees		foraging data (4)
ForagingMap	Simple representation of patches offering available nectar and pollen in the landscape and current foraging activities of the bees	Nectar<br>Pollen<br>All visits	Show BEEHAVE foraging data (4)
SetColour	Choose colour of patch being drawn by the artificial landscape creator; use grey to delete patches	Red<br>Yellow<br>Green<br>Blue<br>Black<br>Grey	Artificial landscape creator (6)
BeeSpecies	Select honeybees or bumblebees	Honeybees<br>Bumblebees	Search options (8)
SearchMode	Options for how a bee chooses its field destination	"colony"<br>"known flowerpatch (individual)"<br>"known flowerpatch (recruitment)"<br>"random location"<br>"visited NLpatch (recruitment)"<br>"furthest location (individual)"<br>"last location (individual)"<br>"mixed strategy (individual)"<br>"mixed strategy (recruitment)"	Search options (8)

The search modes

To define where bees start searching in the landscape after they have left the colony, (described below as the ‘field destination’ i.e. when flightPhase2 begins) the user can choose between SearchModes options. These options take into account possible memory/knowledge of patches already encountered either by the individual bee, or by another bee in the colony.

SearchModes options are:

(i) "colony": no field destination is chosen. On leaving the colony the bee immediately switches to flightPhase 2 to search the area around the hive or colony. This would be a typical choice if the bees have not flown in the landscape before, and do not have information from other bees in the colony.

(ii) "known flowerpatch (individual)": the bee randomly chooses one location that is part of a flower patch already detected by this individual bee. Hence, bigger flower patches are proportionally more likely to be chosen. This could represent the search pattern of bumble bees, where individual bees learn about the landscape, but don't communicate food source locations to their nestmates.

(iii) "known flowerpatch (recruitment)": similar to "known flowerpatch (individual)", but now, a location in any patch that was detected by any bee can become the new destination. Again, the probability of a flower patch to be chosen is proportional to its size. This might occur if bees have information from other nestmates about patch locations (from decoding waggle dances for example).

(iv) "furthest location (individual)": the bee chooses the location which is the furthest from the hive that it has ever been as its new destination. This could represent the search patterns of bees in a poor landscape, with a low density of flower patches, which forces bees to venture further afield.

(v) "visited location (recruitment)": the new destination of a bee is randomly chosen from a location that was previously visited by any bee and is not placed in a lake. (Note that bees can cross lakes when they have a destination). This could be a search mode for honey bees in a landscape with highly transient food sources or if the precision of dances is very low.

(vi) "last location (individual)": the last location a bee has been before it returned to the hive is chosen as destination. This search mode could apply to honey bees and bumble bees with scouts resuming searching at the previously visited location.

(vii) "mixed strategy (individual)": before each scouting trip, one of the above SearchModes that do not require communication ("colony", "known flowerpatch (individual)", "furthest location (individual)", "last location (individual)") is randomly chosen.

(viii) "mixed strategy (recruitment)": before each scouting trip, one of the above colony SearchModes ("colony", "visited NLpatch (recruitment)", "known flowerpatch (recruitment)", "furthest location (individual)" "last location (individual)") is randomly chosen, irrespective if or if not communication is required. The "mixed strategy" search modes take into account that bees will adapt their search behaviour depending on landscape structure and scouting success.

(ix) "random location": any location randomly chosen within the maximal foraging range (MaxForagingRange_m) of the bees, irrespective of whether it has been visited before. This might reflect a situation of an established honeybee colony in a dynamic landscape, where the colony has explored the complete potential foraging area and food sources could regularly appear and disappear in the landscape. It is not included into the "mixed strategy" search modes as it would overpower the effect of the other search modes.

5.1.7. “Slider”

Additionally, the BEESCOUT interface also has several slider options, which allow for a variable to be adjusted on a sliding scale (Table 7).

Table 7 slider variables

“Slider” variable	Definition	Range	Interface
Gridsize	Grid size of the foraging map	0-2000	Show BEEHAVE foraging data (4)
Brushsize	Size of food patch drawn directly from the mouse	0-30	Artificial landscape creator (6)
SetDirection_deg	Set direction of patch from hive	0-360	Artificial landscape creator (6)

LinearisationFactor	Multiplied to the turning angle to allow for a more linear movement	0-1	Bee movement (9)
DisplacementFactor	Factor with which the step length is multiplied	0-10	Bee movement (9)

5.2. Output options

5.2.1. Map display

Foraging activities of the bees are illustrated on a two-dimensional landscape representation, the “foraging map”.

Table 8 map display options (interface section 7)

<table>
<tr><th>Button</th><th>Function</th></tr>
<tr><td>Reset</td><td>resets map to the default display</td></tr>
<tr><td>Grey</td><td>turns all patches grey</td></tr>
<tr><td>Hide all</td><td>hides the bees and the colony, turns all gridcells grey</td></tr>
<tr><td>Patches</td><td>shows/hides food patches & lakes</td></tr>
<tr><td>Color G/B/W</td><td>Turns all gridcells black, white and grey</td></tr>
<tr><td>Labels</td><td>shows/hides patch labels</td></tr>
<tr><td>Patchstats</td><td>shows/hides the Patch statistics (entities keeping track of the detections in each food patch)</td></tr>
<tr><td>model/calc DetProb</td><td>shows the detection probability of the patches, based on modelled detections or on a calculation</td></tr>
<tr><td>Detected</td><td>highlights area of patch detected by bees</td></tr>
<tr><td>History</td><td>shows the chronology of detections</td></tr>
<tr><td>Distribution</td><td>highlights locations that were visited by the bees</td></tr>
<tr><td>Bees</td><td>shows or hides foraging bees
<table>
<tr><th>Colour of bee</th><th>Description</th><th>Flight phase</th></tr>
<tr><td>Green</td><td>Leaving colony toward a destination</td><td>1</td></tr>
<tr><td>White</td><td>Searching the landscape</td><td>2</td></tr>
<tr><td>Orange</td><td>“Looping” behaviour at a patch</td><td>2</td></tr>
<tr><td>Blue</td><td>Returning to colony</td><td>3</td></tr>
</table></td></tr>
<tr><td>Hive</td><td>shows/hides the colony</td></tr>
<tr><td>Borders</td><td>shows/hides the borders of the map</td></tr>
<tr><td>Satellite</td><td>shows satellite image (if available)</td></tr>
<tr><td>Map</td><td>shows the map after colour corrections</td></tr>
<tr><td>Original color</td><td>shows the original colours of the loaded image</td></tr>
<tr><td>Pen</td><td>draws the tracks of the bees</td></tr>
</table>

Erase	deletes the tracks of the bees
Grid	Adds a grid to the foraging map, size determined by "Grid size" slider

5.2.2. Output plots and fields

Live-updating plots and fields show foraging dynamics of bees simulated within BEESCOUT

Table 9 output plots

Plot variable	Description
Covered area [km^2]	Distance covered by all bees in the simulation
Furthest distance [m]	Furthest distance travelled in the simulation
Detection probability per trip	Detection probability per trip, each plot is set to a patch of choice using "Plot1-4" fields

Table 10 output fields

Field variable	Description
Time passed [min]	Time in minutes of model simulation
Time passed [s]	Time in seconds of model simulation
Detected patches	Number of patches detected by scouting bees
Furthest distance [m]	Furthest distance travelled by scouting bees
Min N trips	Minimum number of scouting trips
Mean N trips	Mean number of scouting trips
Date	Current date of the (previously ran) BEEHAVE simulation
Time step	Number time step (time step is every 3 seconds)
Scaling [m/nlPatches]	Size [m] of a Netlogo grid cell

5.2.3. Output file for BEEHAVE

BEESCOUT can also be used to create an input file (*NameOutfile*) for the BEEHAVE colony model.

Table 11 structure of BEESCOUT output file for BEEHAVE

Column	Description
1	day: the day of year (1..365)
2	id: the id of a food patch (0.. N food patches within *MaxForagingRange_m*)
3	oldPatchID: the original id (*who*) of a food patch (i.e. the number shown in the map of the interface) (identical with id, unless food patches are removed due to a limiting *MaxForagingRange_m*)
4	patchType: information about the crop or flowers in the patch (e.g. "OSR" or "RedPatch")
5	distance_m: closest distance [m] of the patch to the hive
6	xcor: distance [m] on x-axis to the hive
7	ycor: distance [m] on y-axis to the hive
8	size_sqm: size of the food patch [m^2]

9	quantityPollen_g: amount of pollen [g] offered at the patch today
10	concentration: sugar (sucrose) concentration [mol/l] of the nectar
11	quantityNectar_l: amount of nectar [l] offered at the patch today
12	calculatedDetectionProb_per_trip: the probability that a scout finds the food patch during a single scouting trip, calculated on the basis of size and distance of the patch
13	modelledDetectionProb_per_trip: the probability that a scout finds the food patch during a single scouting trip as an output of the simulation run
14	nectarGathering_s: handling time [s] for nectar, i.e. the time a bee has to spent in a patch to fill its crop
15	pollenGathering_s: handling time [s] for pollen, i.e. the time a bee has to spent in a patch collect a pollen load

7. List of tables

Table 1 different sections of the BEESCOUT user interface (also highlighted in red above)................. 4
Table 2 pre-defined scenarios available in BEESCOUT 13
Table 3 advanced model initialisations.. 14
Table 4 input fields.. 14
Table 5 switch variables 16
Table 6 chooser variables 17
Table 7 slider variables.. 19
Table 8 map display options 20
Table 9 output plots.. 21
Table 10 output fields 21
Table 11 structure of BEESCOUT output file for BEEHAVE 21
