# Supplementary material for: BEESCOUT: A model of bee scouting behaviour and a software tool for characterizing nectar/pollen landscapes for BEEHAVE
Source: Ecol Modell. 2016 Nov 24;340:126–33. doi: 10.1016/j.ecolmodel.2016.09.013 (PMC5070411; doi:10.1016/j.ecolmodel.2016.09.013)
Supplement: Supplementary file 5 — S5: Methods harmonic radar experiments. [file mmc5.pdf]

MATERIALS AND METHODS

Empirical data

Honey bees

To characterise the searching flights of bees, the tracks of 13 honey bees (*Apis mellifera* L.) from the same hive were recorded with the harmonic radar technique (Riley et al. 1996; Riley & Smith 2002) at Rothamsted Research (Harpenden, Hertfordshire, UK) from 2nd – 16th July 2009. The colony was classified as being "healthy" with only low varroa infestation (daily varroa drop count in June: 0.4) and the individual bees used in the experiment showed a low deformed wing virus load (Wright 2013). The colony was placed at a new location, unfamiliar to the bees (>3 km from their original home location) and the first flight of each naive worker in the new environment was tracked. A bee leaving the hive was caught, equipped with a transponder tag (length: ca. 16mm, weight: ca. 10mg), and then released. After her return, the transponder was removed. The harmonic radar station was placed 266m away from the hive and the harmonic radar signal could be detected within a range of ca. 700m and altitudes between 1-6 m. The location of the bee whilst in flight was recorded every 3 seconds. The recorded tracks had a total length between 28 - 3186 m (mean, s.d.: 812 ± 966 m), a duration between 57 - 3709s (1078 ± 998 s) and the maximal distance flown from the colony during these ‘scouting’ flights was between 10 - 314m (93 ± 103 m). To establish parameters to simulate the flight patterns of the bees, we calculated the average step lengths (i.e. distance between two consecutively recorded locations) and the distribution of turning angles (i.e. change in the flight direction between three consecutively recorded locations) as histogram with a bin width of 10°. The first two recordings after a gap when the radar signal was not picked up (e.g. because the bee was flying too low), were rejected as well as recordings

without any displacement of the bee. In total, 628 valid step lengths and turning angles were obtained. The average displacement per second was 2.96 ± 2.07 m, the distribution of the turning angles is presented in the Supporting Information S4. To capture the typical looping behaviour of bees (Reynolds et al. 2007), number of loops were visually assessed (by counting distinct peaks in plots showing the bees' distance over time) and divided by the total trip duration.

Bumble bees

Step lengths and turning angles for scouting bumble bees (*Bombus terrestris* L.) were derived in the same way as described for honey bees, using the dataset of Osborne et al. 2013. In total, 214 turning angles and step lengths could be calculated from the tracks of 14 naive bumble bees, completing their first trip. The average displacement per second was 3.22 ± 2.24 m, the distribution of the turning angles is presented in the Supporting Information S4.

References

Osborne, J. L., Smith, A., Clark, S. J., Reynolds, D. R., Barron, M. C., Lim, K. S., & Reynolds, A. M. (2013) The ontogeny of bumblebee flight trajectories: From naïve explorers to experienced foragers. PloS ONE, 8, e78681.

Reynolds, A. M., Smith, A. D., Menzel, R., Greggers, U., Reynolds, D. R., & Riley, J. R. (2007) Displaced honey bees perform optimal scale-free search flights. Ecology, 88, 1955-1961.

Riley, J. R., Smith, A. D., Reynolds, D. R., Edwards, A. S., Osborne, J. L., Williams, I. H., Carreck, N.L. & Poppy, G. M. (1996) Tracking bees with harmonic radar. *Nature*, **379**, 29-30.

Riley, J. R., & Smith, A. D. (2002) Design considerations for an harmonic radar to investigate the flight of insects at low altitude. Computers and Electronics in Agriculture, 35, 151-169.

Wright, E. (2013) The effect of pathogens on honeybee learning and foraging behaviour (Doctoral dissertation, University of Warwick, Warwick).
