# Supplementary figures and images for: BEESCOUT: A model of bee scouting behaviour and a software tool for characterizing nectar/pollen landscapes for BEEHAVE
Source: Ecol Modell. 2016 Nov 24;340:126–33. doi: 10.1016/j.ecolmodel.2016.09.013 (PMC5070411; doi:10.1016/j.ecolmodel.2016.09.013)

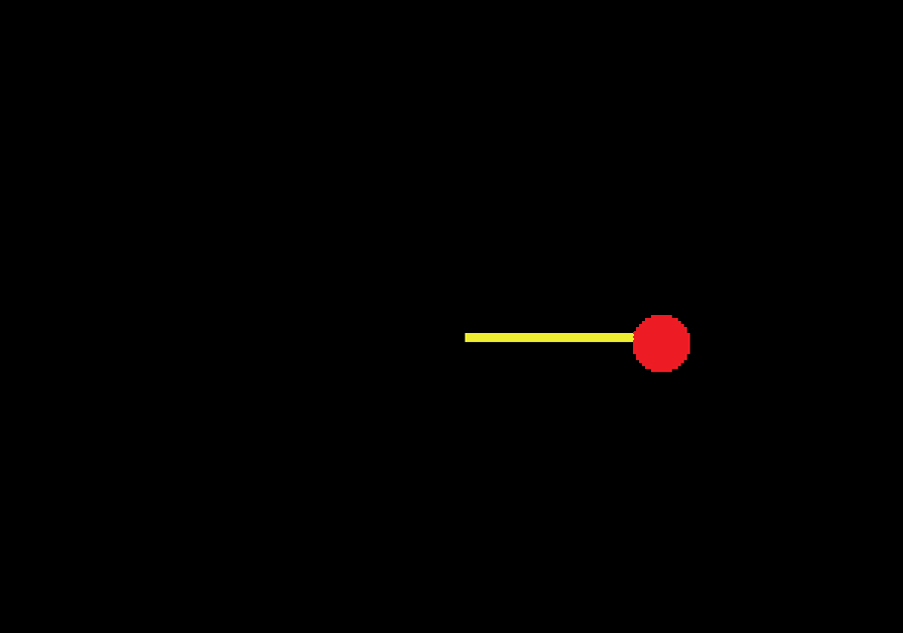

Supplement: Supplementary file 6 — S6(a): Input maps used for the simulations in this publication. [file mmc6.jpg]

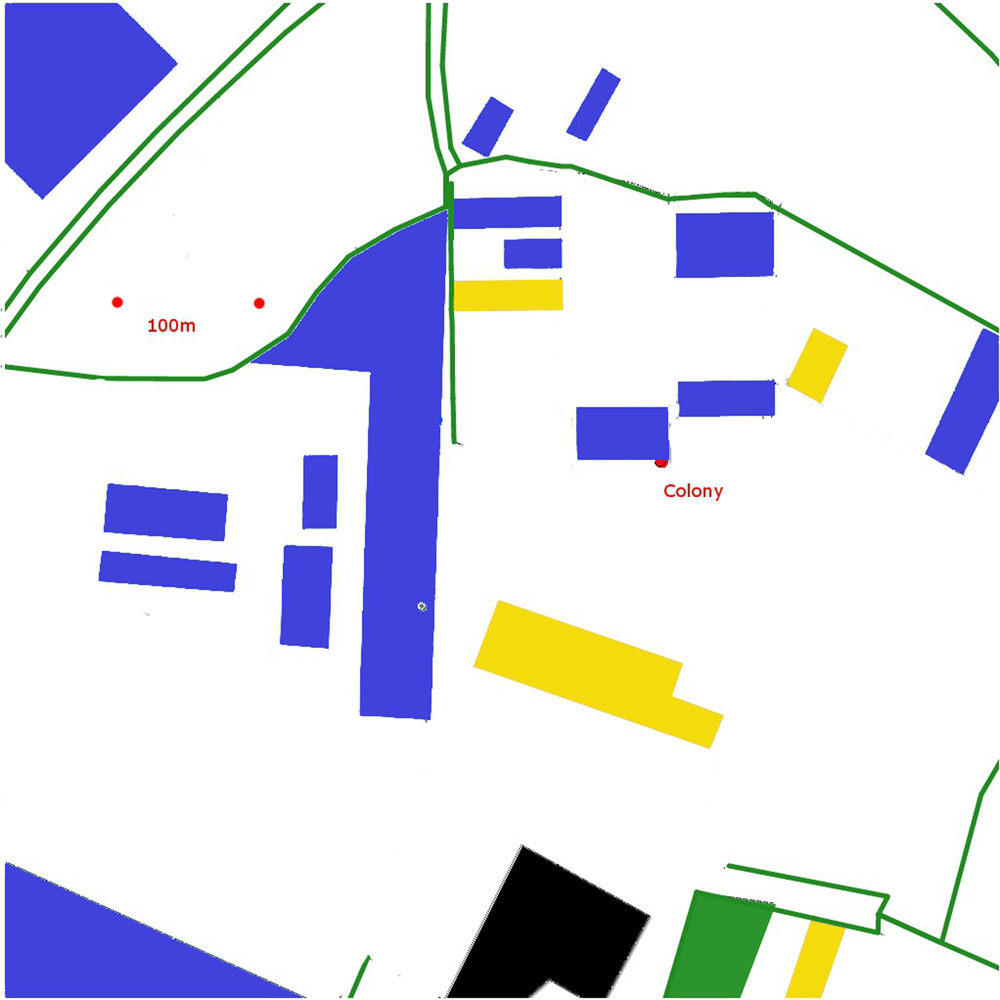

Supplement: Supplementary file 7 — S6(b): Input maps used for the simulations in this publication. [file mmc7.jpg]

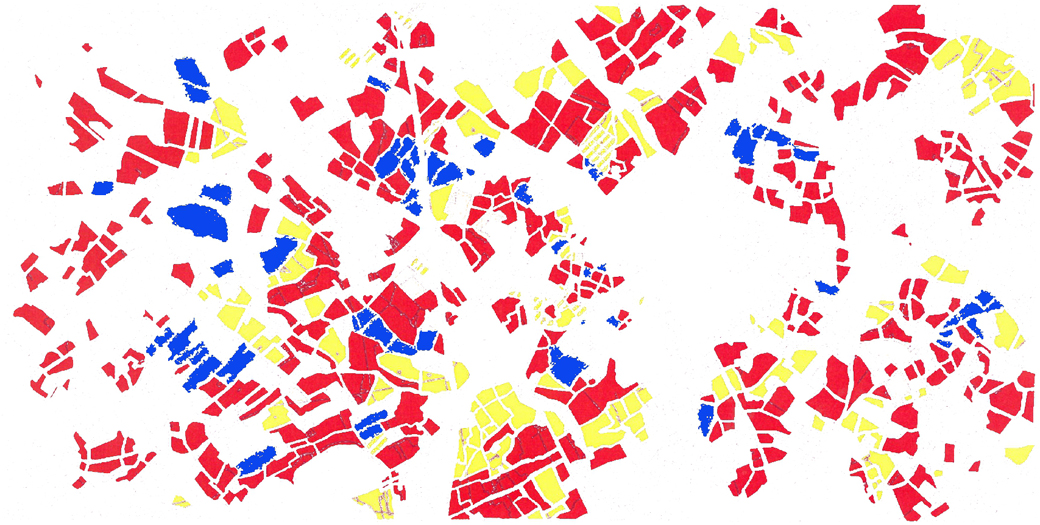

Supplement: Supplementary file 8 — S6(c): Input maps used for the simulations in this publication. [file mmc8.jpg]

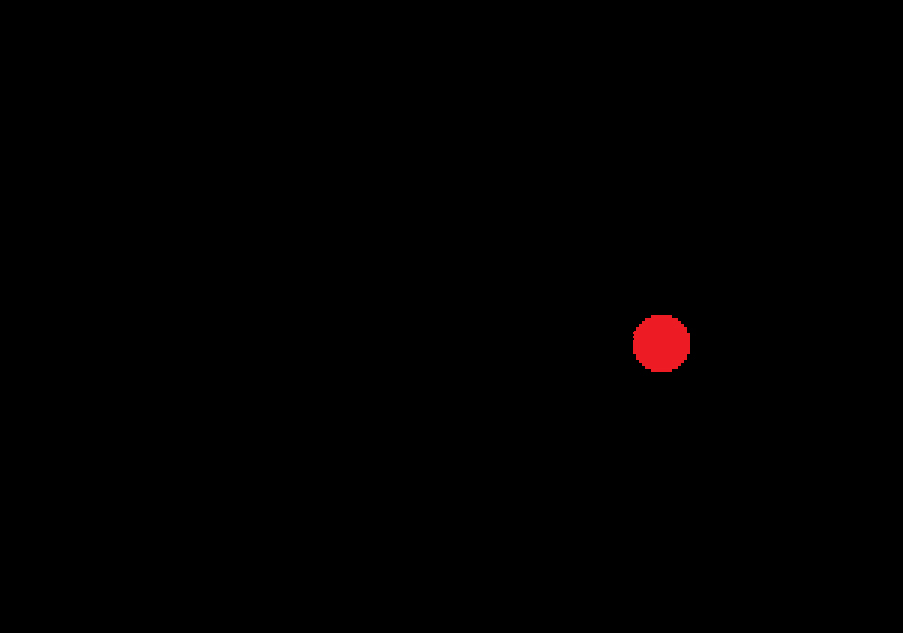

Supplement: Supplementary file 9 — S6(d): Input maps used for the simulations in this publication. [file mmc9.jpg]
