# Supplementary material for: BEESCOUT: A model of bee scouting behaviour and a software tool for characterizing nectar/pollen landscapes for BEEHAVE
Source: Ecol Modell. 2016 Nov 24;340:126–33. doi: 10.1016/j.ecolmodel.2016.09.013 (PMC5070411; doi:10.1016/j.ecolmodel.2016.09.013)
Supplement: Supplementary file 11 — S8: Tables on nectar and pollen production of some crop species. [file mmc11.pdf]

Table A1: Nectar and pollen availability of important crop species: The flowering period of each crop species is given by its start and end date of blooming (day of year). The amount of nectar and pollen per m² in average of each crop species is calculated by its mean number of open flower units per m² and its averaged amount of nectar and pollen produced per flower unit (i.e. single floret, flower head = composition of several single florets) in 24 hours (analogous to maximum nectar and pollen amount per m²). The averaged rage of sugar concentration of each crop species is given in mol / l. The handling times (s) of nectar and pollen represent the time that a bee needs to fill up a full crop volume (50 µl) of nectar and the time a bee needs to collect a full pollen-load (0.015 g). Details of flowering period, # open flowers per m², nectar and pollen amounts per flower unit, sugar concentration, visit time per flower and # visited flowers to collect a full crop volume and to collect a full pollen load are given in Table A2 and A3.

Crop	Flowering		nectar [ml/m2]		pollen [g/m2]		concentration [mol / l]		Handling time [s] nectar		Handling time [s] pollen	
	Start	End	Mean	Max	Mean	Max	Min	Max	Min	Mean	Min	Mean
**Oilseed rape** (*Brassica napus*)	114	136	0.3	1	0.13	0.349	1.3	1.7	213.6	320	180.8	220.5
**Maize** (*Zea mays*)	197	210	0	0	0.752	8.036	-	-	-	-	no data	no data
**Sunflower** (*Helianthus annuus*)	237	264	0.003	0.008	0.108	0.18	0.7	1.8	2949.5	9256.3	75.5	78.9
**Field bean** (*Vicia faba*)	153	182	0.092	0.6	0.0642	0.0945	1.09	1.46	118.2	691.9	225.0	297.5
**White clover** (*Trifolium repens*)	140	242	0.049	0.13	0.0094	0.0141	1.08	1.9	733.3	1630	2084.2	2573.7

Table A2: Literature overview of flowering, nectar and pollen data of important crop species: The duration of flowering is given in days. The daily amount of nectar (µl per flower unit per day) and pollen (mg per flower unit per day) refer to flower heads (composition of several single florets) for sunflower and maize. The nectar and pollen amounts of oilseed rape, white clover and field bean refer to single florets. Sugar concentrations (%) were converted into mol / l using the molar mass of sucrose (M = 342.3 g / mol).

Crop	Period	Flowering [days]	nectar [µl/flower/day]			pollen [mg/flower/day]			Concentration [%]		# Flowers per m² per day		Reference
			Min	Mean	Max	Min	Mean	Max	Min	Max	Min.	Max.	
**Oilseed rape ****<br>(*Brassica napus*)	April - May	22<br>22 – 45[a]	**0.35**[b]	0.55[b]	**0.82**[b]	0.187[d]	0.239[d]	0.292[d]	44[e]	59[e]	543[c]	1194[c]	[a] Radchenko 1964<br>[b] Hedke 2000<br>[c] Blazyte-Cereskiene et al. 2010<br>[d] Von der Ohe et al. 1990<br>[e] Maurizio and Schaper 1994
**Maize *****<br>(*Zea mays*)	June - Sep	14[a]	-	-	-	16[b]	35.3[c]	125.7[c]	-	-	21[b,d]	64[b,d]	[a] Emberlin 1999<br>[b] Percival 1955<br>[c] Miller 1985<br>[d] Olson and Sanders 1988
**Sunflower** [&&]<br>(*Helianthus annuus*)	Aug - Oct	28<br>(19 - 36)[a]	0.22[b,c]	0.81[b,c]	1.39[b,c]	26.6[d]	28.7[a,d]	30[a]	24[b]	61.3[c]	1.5[e]	6[e]	[a] Minckley et al. 1994<br>[b] Hedtke 1998<br>[c] Zajácz et al. 2006<br>[d] Percival 1955<br>[e] AOF 2009
**Field bean**<br>(*Vicia faba*)	June	30<br>30 – 39[a]	0.19[b]	0.86[a]	4.44[c,d]	0.6[e]	-	0.7[c]	6[f]	50[f]	80[a]	135[a]	[a] Brown and Scott 1992<br>[b] Pierre et al. 1996<br>[c] Prabucki et al. 1987<br>[d] Kubisova et al. 1984<br>[e] Percival 1955<br>[f] Osborne et al. 1997
**White clover**<br>(*Trifolium repens*)	May - Oct	102[a]	0.02[b]	0.1[b,c]	0.18[c]	-	0.019[c]	-	37[c,d]	65[b]	247[e]	741[e]	[a] Percival 1950<br>[b] Weaver 1965<br>[c] Aleck 1997, unpubl.<br>[d] Montgomery 1958<br>[e] Free 1993

** The number of open florets of oilseed rape was calculated by the daily number of flowers per plant and the number of plants per m^2. For calculation we used # open flowers / plant per day: (mean = 18.1 and max = 19.9 (Blazyte-Cereskiene et al. 2010)) and # plants / m^2 (mean = 30 and max = 60).

*** Maize provides only large amounts of pollen (no nectar). The pollen amount of maize in average was given in amount of pollen per plant in total (3.5 g per plant in total according to Nowakowski and Morse 1982) and amount of pollen per flower in total (494 mg per flower in total according to Percival 1955). Data of pollen amount per flower per day were not available. Using amount of pollen per plant in total, amount of pollen per flower in total and flowering time per flower (14 days according to Emberlin 1999) we calculated the amount of pollen per flower per day. To calculate the maximum amount of pollen per plant in total we used the maximum number of pollen grains per plant ($50 * 10^6$ pollen grains per plant in total according to Miller 1985) and the weight of one maize pollen grain (0.00025 mg according to Miller 1985). Using the maximum amount of pollen per plant in total, number of flowers per plant (7.1 flowers per plant) and flowering time per flower (14 days) the maximum daily amount of pollen per flower was calculated.

&& The daily nectar amount per head of sunflower was given in mg / head (mean = 0.29 mg / head and max = 0.5 mg / head). For calculation of nectar amount per head in µl we used the Dichte ρ_{nectar} = 360 mg / m l and m_{nectar} = 0.29 mg / head (or 0.5 mg head) to solve the equation $V_{nectar} = m^{*}\rho$.

Table A3: Flower visit data of crop species: Using maximum and mean nectar and pollen amount per flower unit for each crop species we calculated the minimum and mean number of flowers that a bee needs to visit for collecting a full crop volume of nectar (50 µl) and a full pollen load (0.015 g). Number of visited flowers and visit time per flower were used to calculate the handling time of a bee to collect a full crop volume or pollen load.

**Crop**	Visit time per flower [s]		# visited flowers to collect a full crop load		# visited flowers to collect a full pollen load		References
	Min	Mean	Min	Mean	Min	Mean	
**Oilseed rape** (*Brassica napus*)	-	3.52[a]	60.97	90.91	51.37	62.63	[a] Picard-Nizou et al. 1995
**Sunflower** (*Helianthus annuus*)	82[a]	151[b]	35.97	61.73	0.5	0.52	[a] Free 1993<br>[b] Fell 1986
**Field bean** (*Vicia faba*)	10.5[a]	11.9[a]	11.26	58.14	21.43	25	[a] Free 1962
**White clover** (*Trifolium repens*)	2.64[a]	3.26[a]	277.78	500	-	789.47	[a] Weaver 1965

References

Aleck, R 1997 (unpubl.). A comparison of nectar production in seven varities of white clover.

AOF. 2009. Australian Oilseeds Federation (Online). URL: http://www.australianoilseeds.com, (accessed 2015).

Blažytė-Čereškienė, L. et al. 2010. Honey bee foraging in spring oilseed rape crops under high ambient temperature conditions. Zemdirbyste-Agriculture 97: 61-70.

Brown, B. and Scott, R.R. 1992. An assessment of *Vicia faba* and *Trifolium pratense* as forage crops for *Bombus hortorum*. New Zealand Entomologist 15: 42-47.

Emberlin, J. 1999. A report on the dispersal of maize pollen. Research paper commissioned by the Soil Association, http://www.soilassociation.org

Fell, R.D. 1986. Foraging behaviors of *Apis mellifera* L. and *Bombus spp*. on oilseed sunflower (*Helianthus annuus* L.). Journal of the Kansas Entomological Society 59: 72-81.

Free, J. B. 1962. The behaviour of honeybees visiting field beans (*Vicia faba*). J. Anim. Ecol. 31: 497-502.

Free JB 1993. Insect Pollination of Crops (2nd ed). Academic Press: London, UK.

Hedtke, C. 1998. Die Sonnenblume – ihre Bedeutung als Bienenweide. Deutsches Bienenjournal 11: 459-462.

Hedtke, C. 2000. Nektar- und Pollenproduktion transgener und konventioneller Rapsblüten. Deutsches Bienenjournal 3: 109-110.

Maurizio, A. and Schaper, F. 1994. Das Trachtpflanzenbuch. Nektar und Pollen – die wichtigsten Nahrungsquellen der Honigbiene. Ehrenwirth: München.

Miller PD, 1985. Maize Pollen: Collection and Enzymology. Chapter 45, pp 279-282. In: Sheridan WF (ed.). 1985. Maize for Biological Research. A Special Publication of the Plant Molecular Biology Association, USA.

Minckley, R.L. et al. 1994. Behavior and phenology of a specialist bee (*Dieunomia*) and sunflower (*Helianthus*) pollen availability. Ecology 75: 1406-1419.

Montgomery, B. E. 1958. Preliminary studies of the composition of some Indiana nectars. Proc. Indiana Acad. Sci. 68: 159-163.

Nowakowski, J. and Morse, R.A. 1982. The behavior of honey bees in sweet corn fields in New York State. American Bee Journal 122: 13–16.

Olson, R.A. and Sanders, D.H. 1988. Maize production. In: Sprague, G.F. and Dudley, J.W. Corn and corn improvement. Madison : American Society of Agronomy, Cap.11, p.639-686.

Osborne, J.L. et al. 1997. Nectar and flower production in *Vicia faba* L (field bean) at ambient and elevated carbon dioxide. Apidologie 28: 43-55.

Percival, M. S. 1950. Pollen presentation and pollen collection.New Phytol. 49: 40-63.

Percival, M. S. (1955). The presentation of pollen in certain angiosperms and its collection by *Apis mellifera*. New Phytol. 54: 353-68.

Picard-Nizou, A.L. et al. 1995. Foraging behaviour of honey bees (*Apis mellifera* L.) on transgenic oilseed rape (*Brassica napus* L. *var. oleifera*). Transgenic Research 4: 270-276.

Pierre, J. et al. 1996. Comparative study of nectar secretion and attractivity to bees of two lines of spring-type faba bean (*Vicia faba* L var *equina* Steudel). Apidologie 27: 65-75.

Prabucki, J., Chuda-Mickiewicz, J. B. and Woyke, H. 1987. The influence of bees on the crop of broad bean seeds *(Vicia faba* L. var. *major).* The 31st International Apicultural Congress, Warsaw, Poland, 403-109. Bucharest: Apimondia Publishing House.

Radchenko, T. G. 1964. The influence of pollination on the crop and the quality of seed of winter rape. Bdzhil'nitstvo 1: 68-74.

Von der Ohe, W., Dustmann, J.H. and von der Ohe, K. 1990. Untersuchung von Winterrapshybriden und Liniensorten auf Unterschiede im Pollengehalt. Deutsches Bienenjournal 4: 145-146.

Weaver, N. 1965. Foraging behaviour of honeybees on white clover. Insectes Soc. 12: 231-240.

Zajácz, E. et al. 2006. Nectar production of some sunflower hybrids. Journal of Apicultural Science 50: 109-113.
