# Supplementary material for: BEESCOUT: A model of bee scouting behaviour and a software tool for characterizing nectar/pollen landscapes for BEEHAVE
Source: Ecol Modell. 2016 Nov 24;340:126–33. doi: 10.1016/j.ecolmodel.2016.09.013 (PMC5070411; doi:10.1016/j.ecolmodel.2016.09.013)
Supplement: Supplementary file 12 — S9: Comparison of modelled and empirical bumblebee flight tracks. [file mmc12.pdf]

Comparison empirical and modelled bumblebee flight paths

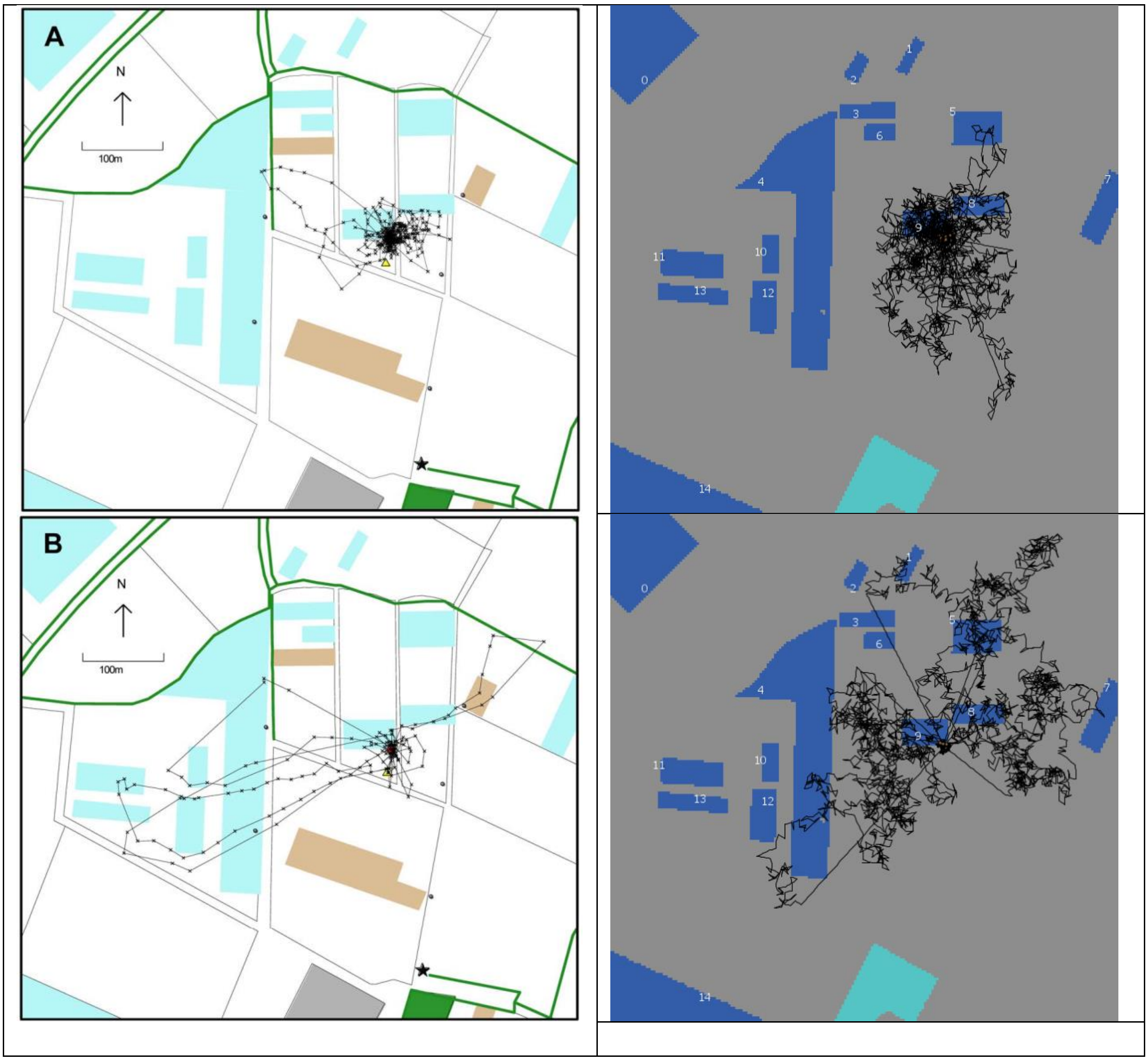

Empirical (left, from Osborne et al. 2013) and an example of modelled (right) tracks of bumble bees ($N_{Simulations}$ = 1). A) Bees on their first scouting trip (n = 14), B) bees on 2nd and 3rd trips (modelled: only 3rd trip)(n = 6). Experimental bees were more directed in their flights – likely as some of them began to forage and stopped searching- whilst the model shows continued searching behaviour – but the flight ranges of modelled and empirical data match well.
