# Supplementary material for: BEESCOUT: A model of bee scouting behaviour and a software tool for characterizing nectar/pollen landscapes for BEEHAVE
Source: Ecol Modell. 2016 Nov 24;340:126–33. doi: 10.1016/j.ecolmodel.2016.09.013 (PMC5070411; doi:10.1016/j.ecolmodel.2016.09.013)
Supplement: Supplementary file 13 — S10: Sensitivity analysis. [file mmc13.pdf]

Sensitivity analysis

We performed a sensitivity analysis for a selection of important parameters, based on the default setting for honeybees and varying one parameter at a time. Simulations took place in an artificial and highly simplified landscape, with only a single, approx. circular flower patch (radius: 96m; shortest distance colony - patch: 415m).
The default search mode for honeybees is "known flowerpatch (recruitment)". In a landscape with only a single flower patch, this results in all scout bees choosing this patch as the "field destination" once it is deteced for the first time (i.e. they head in a linear vector flight towards the patch and then switch to the small scaled search mode). For this reason, we stopped the simulations as soon as the patch was detected and used the time until first detection (mean ± s.d., N = 30) as an output, rather than the actual detection probability.

Default values are shown as open circles, x represents simulations where the patch was not detected in any of the 30 runs.

Control

Time [s] till first detection in the control runs: **2135.7 ± 1813.1**

DisplacementFactor (default value: 1)

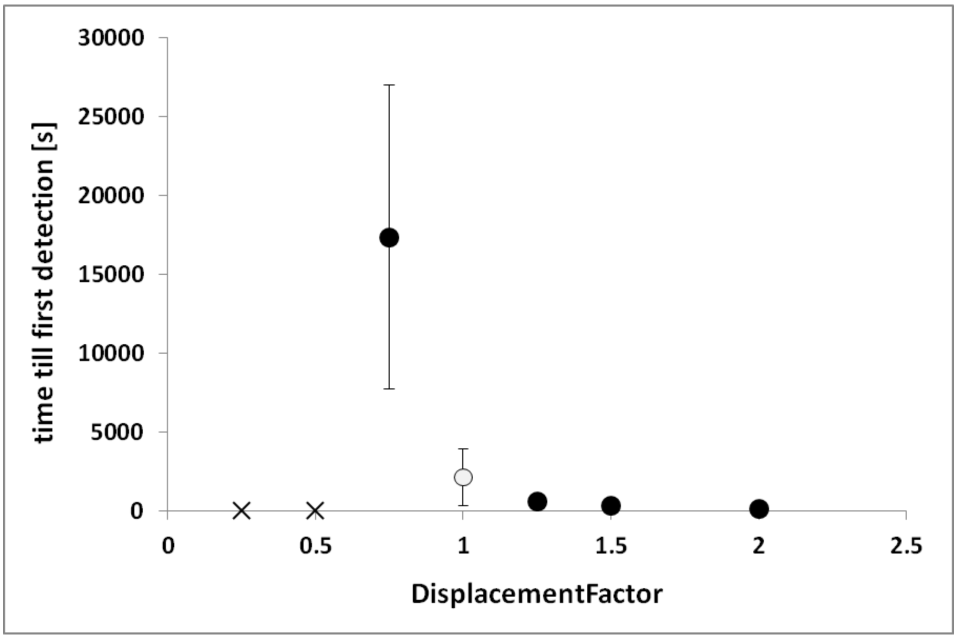

(DisplacementFactor modifies the bees step width, i.e. a value of 2 doubles the speed)

LinearisationFactor (default value: 1)

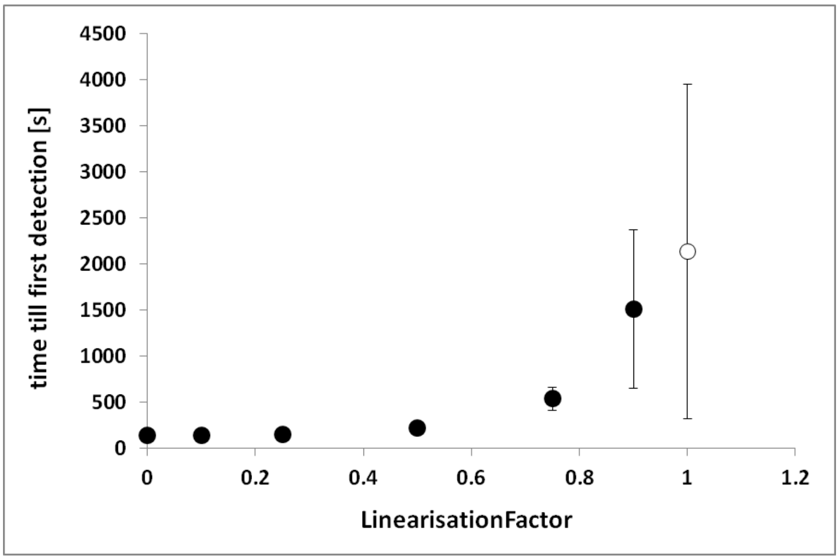

(Values smaller than 1 make the movement of the bees more linear resulting in a straight line for a value of 1)

RandomWalk (default value: false)

Time [s] till first detection if RandomWalk = true: **12760.5 ± 10291.2** (control: 2135.7 ± 1813.1)

(If RandomWalk is true, the bees' direction is not chosen from the distribution of the experimentally determined turning angles but randomly chosen from a uniform distribution of angles)

N_Bees (default value: 10000)

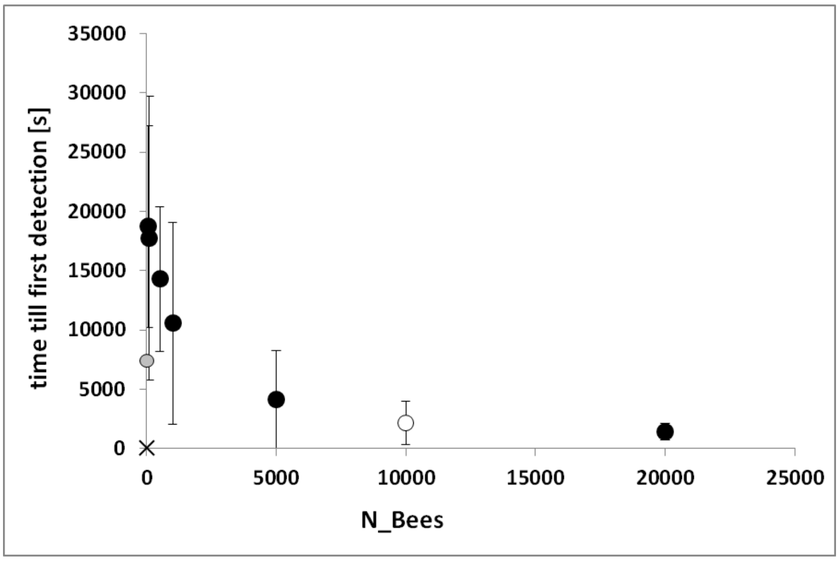

(The number of bees. Values tested: 1; 10; 50; 100; 500; 1000; 5000; 10000; 20000. The grey circle (N_bees = 10) is based on only a single run where the patch was detected)

TripDuration_s (default value: 1020)

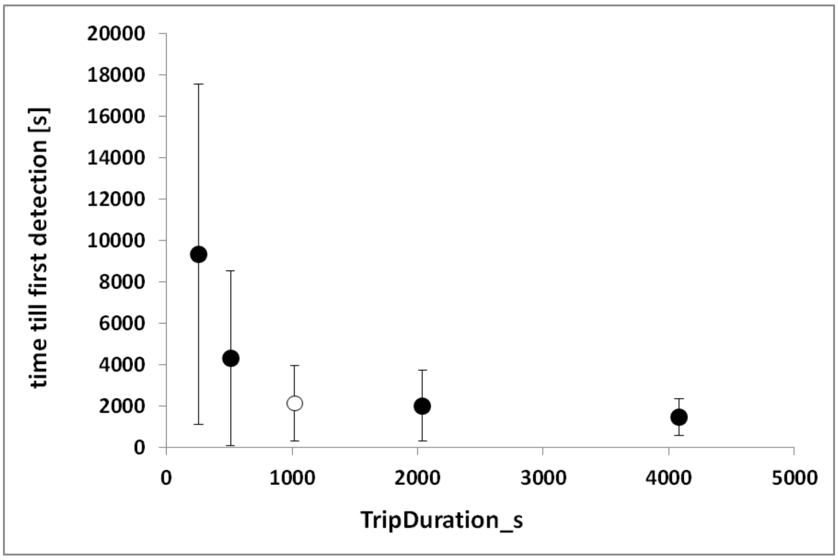

(TripDuration_s is the average time a bee spend on a scouting trip before it turns back to the colony)

TurnToDestinationProb (default value: 0.02)

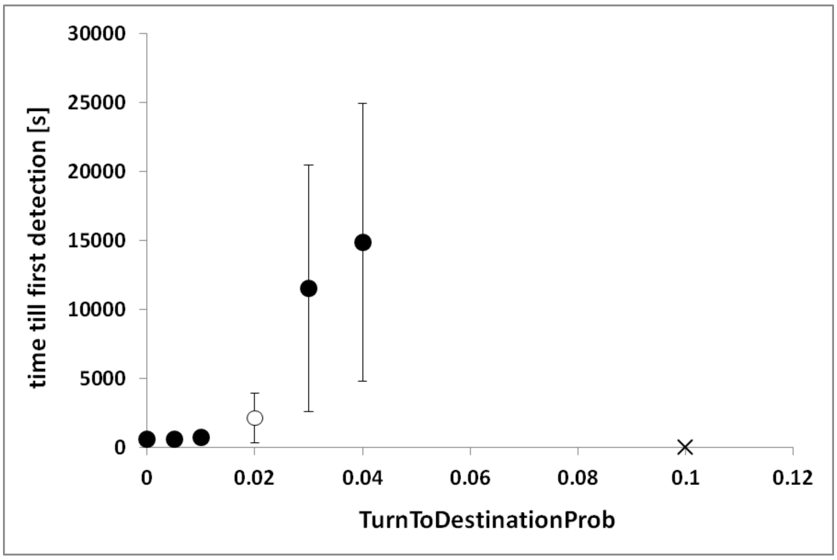

(TurnToDestinationProb is the probability for each time step that bee during its small scaled search flight (i.e. in flightPhase 2) returns to its current 'field destination'. Higher probabilities result in increased looping behaviour).

SearchMode (default: "known flowerpatch (recruitment)")

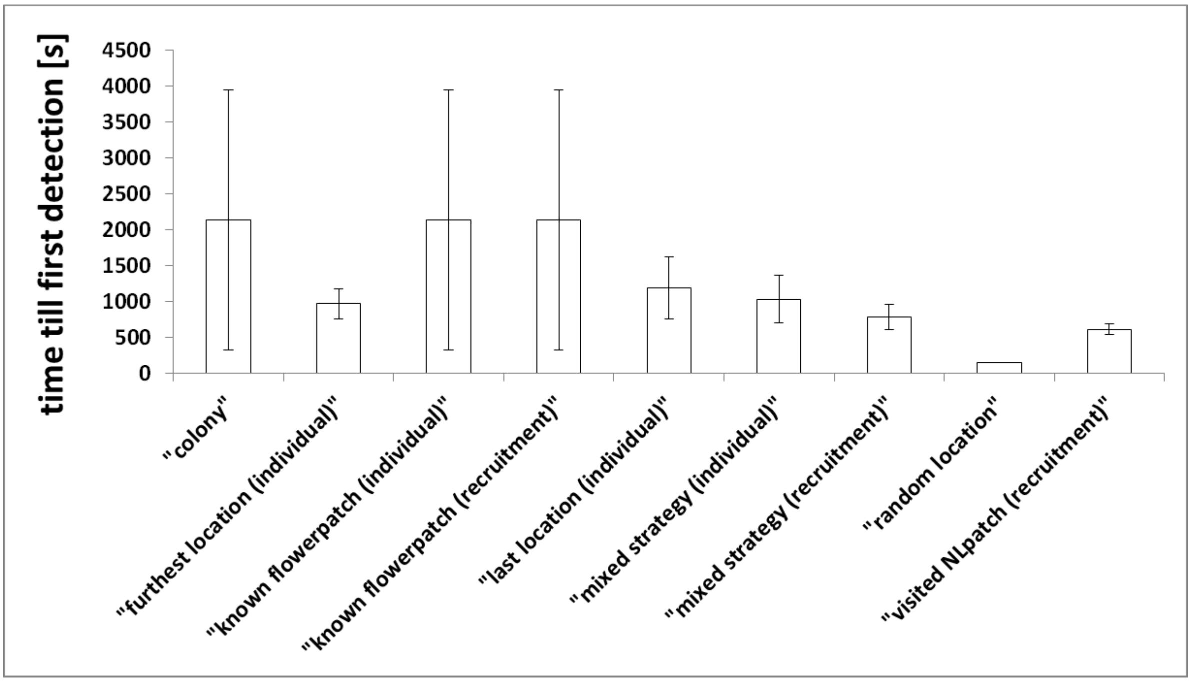

(The search modes "known flowerpatch (individual)" and "known flowerpatch (recruitment)" differ from the search mode "colony" only if at least one patch is "known" to one or all bees. As this analysis was stopped after the first detection of the patch, the results are identical for these three search modes.)

BeeSpecies "Bumblebees" (default: "Honeybees")

When BeeSpecies is set to "Bumblebees" (i.e. the turning angles are chosen from the distribution for bumblebees but all other parameters are still set to the default values for honeybees, i.e. N_Bees = 10000, SearchMode = "known flowerpatch (recruitment)", TripDuration_s = 1020) then the first patch detection takes place after **13149.8 ± 7993.2** seconds (control: 2135.7 ± 1813.1)

When running the model under the full the bumblebee setting (SearchMode = "known flowerpatch (individual)", N_Bees = 30, TripDuration_s = 600), the patch was not detected in any of the 30 runs.
